# Structure and Development of Flowers and Inflorescences in *Burmannia* (Burmanniaceae, Dioscoreales)

**DOI:** 10.3389/fpls.2022.849276

**Published:** 2022-03-18

**Authors:** Sophia V. Yudina, Alexander Kocyan, Ba Vuong Truong, Nikolay A. Vislobokov, Dmitry F. Lyskov, Maxim S. Nuraliev, Margarita V. Remizowa

**Affiliations:** ^1^Department of Higher Plants, Faculty of Biology, M.V. Lomonosov Moscow State University, Moscow, Russia; ^2^Joint Russian-Vietnamese Tropical Scientific and Technological Center, Hanoi, Vietnam; ^3^Department of Plant and Microbial Biology, Botanical Museum, University of Zurich, Zurich, Switzerland; ^4^Department of Biological Resources, Institute of Tropical Biology, Vietnam Academy of Science and Technology, Ho Chi Minh City, Vietnam

**Keywords:** floral anatomy, gynoecium, gynostegium, monochasium, placentation, vasculature

## Abstract

Species of the genus *Burmannia* possess distinctive and highly elaborated flowers with prominent floral tubes that often bear large longitudinal wings. Complicated floral structure of *Burmannia* hampers understanding its floral evolutionary morphology and biology of the genus. In addition, information on structural features believed to be taxonomically important is lacking for some species. Here we provide an investigation of flowers and inflorescences of *Burmannia* based on a comprehensive sampling that included eight species with various lifestyles (autotrophic, partially mycoheterotrophic and mycoheterotrophic). We describe the diversity of inflorescence architecture in the genus: a basic (most likely, ancestral) inflorescence type is a thyrsoid comprising two cincinni, which is transformed into a botryoid in some species via reduction of the lateral cymes to single flowers. *Burmannia oblonga* differs from all the other studied species in having an adaxial (vs. transversal) floral prophyll. For the first time, we describe in detail early floral development in *Burmannia*. We report presence of the inner tepal lobes in *B. oblonga*, a species with reportedly absent inner tepals; the growth of the inner tepal lobes is arrested after the middle stage of floral development of this species, and therefore they are undetectable in a mature flower. Floral vasculature in *Burmannia* varies to reflect the variation of the size of the inner tepal lobes; in *B. oblonga* with the most reduced inner tepals their vascular supply is completely lost. The gynoecium consists of synascidiate, symplicate, and asymplicate zones. The symplicate zone is secondarily trilocular (except for its distal portion in some of the species) without visible traces of postgenital fusion, which prevented earlier researchers to correctly identify the zones within a definitive ovary. The placentas occupy the entire symplicate zone and a short distal portion of the synascidiate zone. Finally, we revealed an unexpected diversity of stamen-style interactions in *Burmannia*. In all species studied, the stamens are tightly arranged around the common style to occlude the flower entrance. However, in some species the stamens are free from the common style, whereas in the others the stamen connectives are postgenitally fused with the common style, which results in formation of a gynostegium.

## Introduction

*Burmannia* L. is one of ca. 16 angiosperm genera which contain both photosynthetic and mycoheterotrophic species (Merckx et al., 2013). Some of these genera have only a single mycoheterotrophic species. *Burmannia* is among those few genera where species with both lifestyles are well represented: 1/3 of ca. 60 its species are mycoheterotrophic (Maas-van de Kamer, 1998; Govaerts et al., 2011; Merckx et al., 2013). Phylogenetic investigations have demonstrated that loss of photosynthetic activity has independently occurred at least four times within *Burmannia* (Merckx et al., 2006, 2008). Moreover, only a few of photosynthetic species (exhibiting green plant coloration) are fully autotrophic; the others are characterized by very small leaves and believed to be partially mycoheterotrophic (Merckx et al., 2013). Thus, *Burmannia* is one of the few species-diverse angiosperm lineages with different lifestyles, which makes this genus highly relevant for investigations of evolution of mode of nutrition and related diversity and evolution of morphological characters.

Despite diverse nutrition pathways of mycoheterotrophic plants, modes of reproduction are the same as in autotrophic plants: sexual pathway occurs in all mycoheterotrophic lineages whereas the asexual way is present only in some groups (Leake, 1994). Mycoheterotrophic plants are predominantly biotically pollinated, and often exhibit spectacular floral appearance and remarkable morphology due to their pollination mechanisms (Leake, 1994). This is also the case for the entire genus *Burmannia* (including its photosynthetic species), which shows a set of elaborations of floral structure. Flowers of *Burmannia* demonstrate almost typical trimerous monocot groundplan; they are bisexual, actinomorphic, characterized by a small perianth of six tepals in two whorls and a syncarpous gynoecium with an inferior ovary. The androecium of *Burmannia* consists of only three stamens inserted opposite inner tepals, which is a relatively rare condition for monocots (Maas et al., 1986; Rübsamen, 1986; Maas-van de Kamer, 1998; Zhang, 1999; Caddick et al., 2000; Merckx et al., 2013). The tepal lobes are placed on a long tubular floral tube (termed as a hypanthium by Caddick et al., 2000), which is three- (or six-) ribbed to broadly three-winged. The perianth ribs/wings run from the ovary wall to the apex of the floral tube, so that the boundary between the ovary and the floral tube is not evident from the outside. The inner tepal lobes are smaller than the outer ones, and sometimes they are absent. The stamens are inserted on the inner surface of the floral tube close to its apex. They are believed to represent the inner whorl of the androecium, whereas the outer whorl is completely reduced. The stamen connective is broad, separating the two thecae, and provided with one basal and two apical appendages. Stamens contact the stigmas (commonly described as connivent with them), occluding the orifice of the floral tube. The three styles are initially free and fuse during development into a common style (termed as stylar column by Caddick et al., 2000), which has a central canal and three free stylar branches (Zhang, 1999; Caddick et al., 2000). The ovary is usually considered to be trilocular, with axile placentation (Maas et al., 1986; Maas-van de Kamer, 1998; Merckx et al., 2013), but in some cases it is trilocular proximally and unilocular distally (Pai, 1966; Rao, 1969; Zhang, 1999). In some species, septal nectaries are present in the form of tiny clefts with simple shape (Pai, 1966; Rao, 1969; Maas et al., 1986; Rübsamen, 1986; Caddick et al., 2000).

Although flowers of *Burmannia* have been attracting the attention of morphologists for more than a century, some important gaps in understanding their structure still exist, as well as the contradictions between the observations made by different authors. In addition, the insufficient knowledge of floral morphology complicates taxonomy of *Burmannia* at a species level. For instance, the shape of margins of the tepal lobes (single vs. double margins) is used to distinguish some of the species, but has never been examined for the remaining species. Then, *B. lutescens* Becc. is accepted by Zhang (1999) in a wide sense, and described to be an extremely polymorphic species, whose most striking variation is presence or absence of the inner tepal lobes. However, the presence vs. absence of a floral whorl is usually far beyond the intraspecific variation in monocots (Remizowa et al., 2010b). Such striking discrepancies and gaps require special clarification.

Several species of *Burmannia* have been studied with respect to floral anatomy and vasculature. Rübsamen (1986) presented detailed data on *B. bicolor* Mart., *B. grandiflora* Malme, *B. longifolia* Becc. and *B. vaupesiana* Benthem and Maas, along with some additional information for several other species. Pai (1966) provided results on *B. pusilla* (Miers) Thwaites, and Rao (1969) on *B. disticha* L. and *B. nepalensis* (Miers) Hook.f. A brief description of floral vasculature in *B. disticha* is also given by Zhang (1999). Finally, certain anatomical data were obtained by Ernst and Bernard (1911) and Bernard and Ernst (1914) for *B. lutescens* [as *B. candida* (Blume) Engl., nom. illeg.] and *B. championii* Thwaites (including *B. tuberosa* Becc., which these authors considered to be a distinct species).

Observations of floral development are available for only a few species of *Burmannia*. Rübsamen (1986) investigated *B. bicolor*. Three species were studied by Caddick et al. (2000), of which only *B. coelestis* D. Don is illustrated; the study includes a description of only some aspects of floral development, and contains very little data on early development and no information on ovary formation. Zhang (1999) provided a description for *B. disticha* and several additional species, based on sections of flowers. None of these reports provides thorough developmental series. SEM data were obtained only for the stage of perianth initiation and the late stages with all floral parts already formed.

Inflorescences of *Burmannia* received less attention and thus are poorly understood. The characters of inflorescence structure are mainly aimed in species identification; they have never been a subject to a detail morphological analysis. The inflorescences are usually described as single-flowered to many-flowered terminal cymes. The exact structure of cymes is unclear. They are described either as a bifurcate cincinnus (e.g., Maas et al., 1986; Maas-van de Kamer, 1998; Zhang, 1999) or as a double-rhipidium (Dahlgren et al., 1985). These two interpretations differ by position of floral prophylls (lateral vs. median).

This paper is an attempt to develop a comprehensive view on the inflorescence and floral structure in *Burmannia*. We provide new data on eight Asian species, of which *B. disticha* and *B. longifolia* are fully autotrophic, *B. chinensis* Gand. and *B. coelestis* are partially mycoheterotrophic and *B. championii*, *B. itoana* Makino, *B. lutescens* and *B. oblonga* Ridl. are fully mycoheterotrophic. Detailed field photographs of the specimens under study are provided by Nuraliev et al. (2018, 2022). We employ observations of intact inflorescences, serial microtome sections of preanthetic flower buds as well as the developing flowers, and SEM observations of flowers at various developmental stages. Our goal is to obtain the following novel knowledge for the genus: (1) to reveal the diversity of inflorescence architecture; (2) to describe and illustrate the structure of the ovary, and establish the gynoecium zonation and the placentation type; (3) to specify the interactions between the stamens and the common style; (4) to find the limits of variation of floral vasculature, and test its correlations with gross morphology of the flower. We provide a comparison of floral structure in *Burmannia* with that of the other taxa of Dioscoreales. Finally, we use our results to resolve certain terminological issues, particularly regarding the floral tube and parts of the gynoecium.

## Materials and Methods

Information on the specimens examined, including details of vouchers and number of sectioned flowers, is presented in Table 1. Whole plants and inflorescences were fixed and stored in 70% ethanol.

**TABLE 1:** Voucher specimens for the examined material, and the investigations performed.

Species	Voucher	Place and date of collection	Investigation
*Burmannia championii* Thwaites	*A. Kocyan AK811* (Z)	Malaysia, Sarawak, 2009	Floral anatomy (one pre-anthetic flower, one underdeveloped flower)
*Burmannia chinensis* Gand.	*Truong Ba Vuong, Dang Van Son BV999* (spirit material: MW, VNM)	Vietnam, Tay Ninh Province, Tan Bien District, Lo Go Xa Mat National Park, grassland, half open canopy, 21.11.2020	Inflorescence morphology, floral organography, anatomy (one pre-anthetic flower) and development
*Burmannia coelestis* D.Don	*M. S. Nuraliev, I. A. Savinov, A. B. Shipunov, N. A. Vislobokov 2736* (spirit material: IBSC, MW)	Brunei, Brunei-Muara District, Berakas Forest Reserve, disturbed shrubby forest on sand dunes, 4°59′36″N 114°55′38″E, 30 m, 03.07.2019	Inflorescence morphology, floral organography, anatomy (one pre-anthetic flower, one underdeveloped flower) and development
*Burmannia coelestis* D.Don	*Truong Ba Vuong, Dang Van Son BV1000* (spirit material: MW, VNM)	Vietnam, Tay Ninh province, Lo Go Xa Mat National Park, grassland, open canopy, 21.11.2020	Inflorescence morphology, floral organography and development
*Burmannia disticha* L.	*N. A. Vislobokov 19039* (spirit material: MW)	Vietnam, Lao Cai Province, Bat Xat District, Bat Xat Nature Reserve, 4 km SSE of Y Ty village, disturbed forest, 22°37.539′N 103°37.658′E, 1838 m, 04.06.2019	Inflorescence morphology, floral organography, anatomy (one pre-anthetic flower) and development
*Burmannia itoana* Makino	*M. S. Nuraliev 2444* (spirit material: IBSC, MW)	Vietnam, Quang Nam Province, Nam Giang District, Song Thanh Nature Reserve, forest, river bank, 15°34′12″N 107°22′39″E, 1050 m, 30.04.2019	Inflorescence morphology, floral organography, anatomy (one pre-anthetic flower) and development
*Burmannia longifolia* Becc.	*A. Kocyan SAN150269* (Z)	Malaysia, Sabah	Floral anatomy (one pre-anthetic flower)
*Burmannia lutescens* Becc.	*M. S. Nuraliev, A. N. Kuznetsov, S. P. Kuznetsova 1657* (spirit material: IBSC, MW)	Vietnam, Kon Tum province, Kon Plong district, Thach Nham protected forest, 17 km N of Mang Den town, in the forest, on slope, 14°45′05″ N 108°18′25″E, 1150 m, 06.06.2016	Floral organography and development
*Burmannia lutescens* Becc.	*M. S. Nuraliev, D. F. Lyskov NUR 3120* (spirit material: MW)	Vietnam, Phu Yen Province, Song Hinh District, Song Hinh Municipality, Song Hinh Protected Forest, forest on slope, near trail, 12°48′00″N 109°00′50″E, 360 m, 13.01.2021	Inflorescence morphology, floral organography, anatomy (one pre-anthetic flower) and development
*Burmannia oblonga* Ridl.	*N. A. Poyarkov s.n.* (spirit material: IBSC, MW)	Thailand, Chanthaburi Province, Khao Soi Dao Wildlife Sanctuary, Khao Soi Dao Mt., 12°54′16″N 102°11′49″E, 1156 m, 19.11.2016	Inflorescence morphology, floral organography, anatomy (one pre-anthetic flower) and development
*Burmannia oblonga* Ridl.	*M. S. Nuraliev, D. F. Lyskov NUR 3160* (spirit material: MW)	Vietnam, Dak Nong province, Dak Glong District, Dak Plao municipality, Ta Dung National Park, 38 km ESE of Gia Nghia city, forest, near small stream, 11°51′21″N 108°00′21″E, 1080 m, 20.01.2021	Inflorescence morphology, floral organography, anatomy (one pre-anthetic flower) and development
*Burmannia oblonga* Ridl.	*S. V. Yudina, M. S. Nuraliev 1* (spirit material: IBSC, MW)	Vietnam, Dak Lak province, Lak district, Bong Krang municipality, Chu Yang Sin National Park, in the forest, rocky river bank, 12°24′05″N 108°21′08″E, 1001 m, 20.05.2019	Inflorescence morphology, floral organography

**FIGURE 1:**
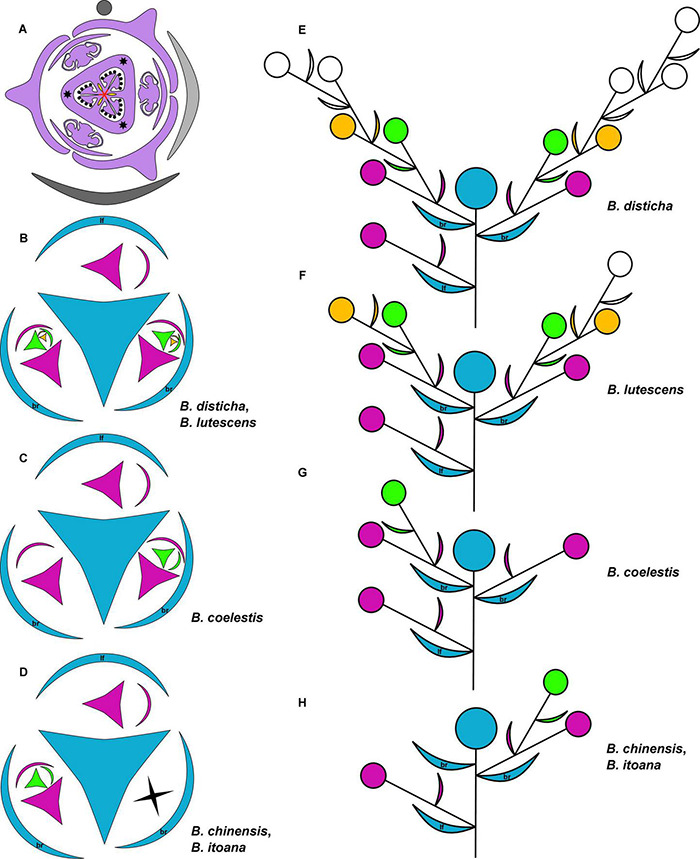
**(A)** Diagram of lateral flower of the species of *Burmannia* studied here; asterisks indicate position of styles; red lines: postgenital fusion; yellow areas: septal nectaries. **(B–H)** Diagrams of the inflorescence structure of *Burmannia*: groundplan, with shape of flower indicating position of perianth wings and outer tepals **(B–D)**; lateral view **(E–H)**. br, involucral bract; lf, stem leaf.

**FIGURE 2:**
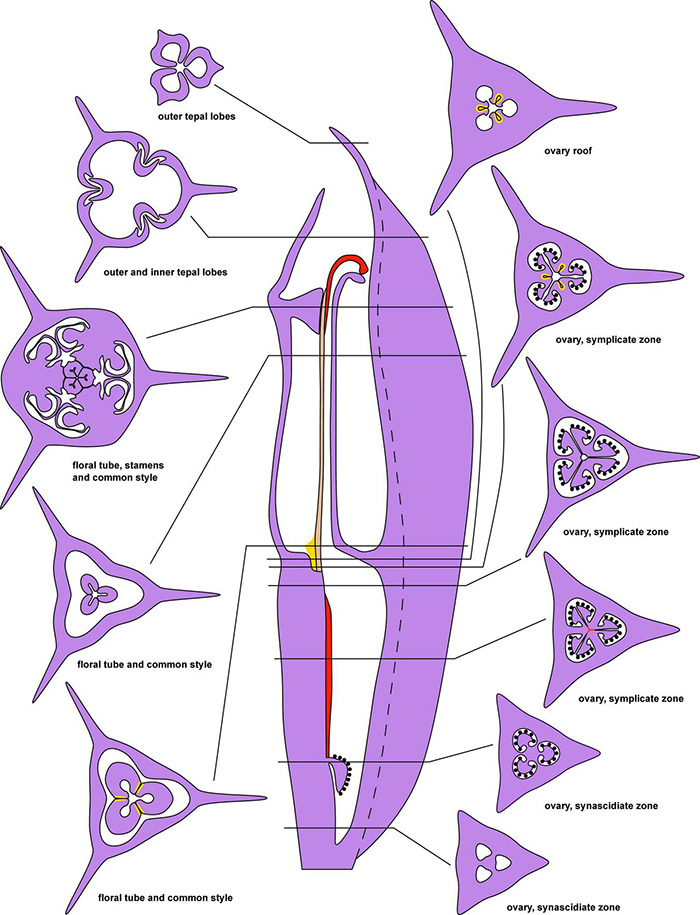
Diagrams of longitudinal section and transverse sections of a flower of *Burmannia* exemplified by *B. disticha*. Dashed lines indicate boundary of perianth wing; red areas and lines: postgenital fusion within carpels; brown area: postgenital fusion between carpels; yellow areas: septal nectaries.

Diagrams (Figures 1, 2) were prepared using Inkscape, v.0.92.

**FIGURE 3:**
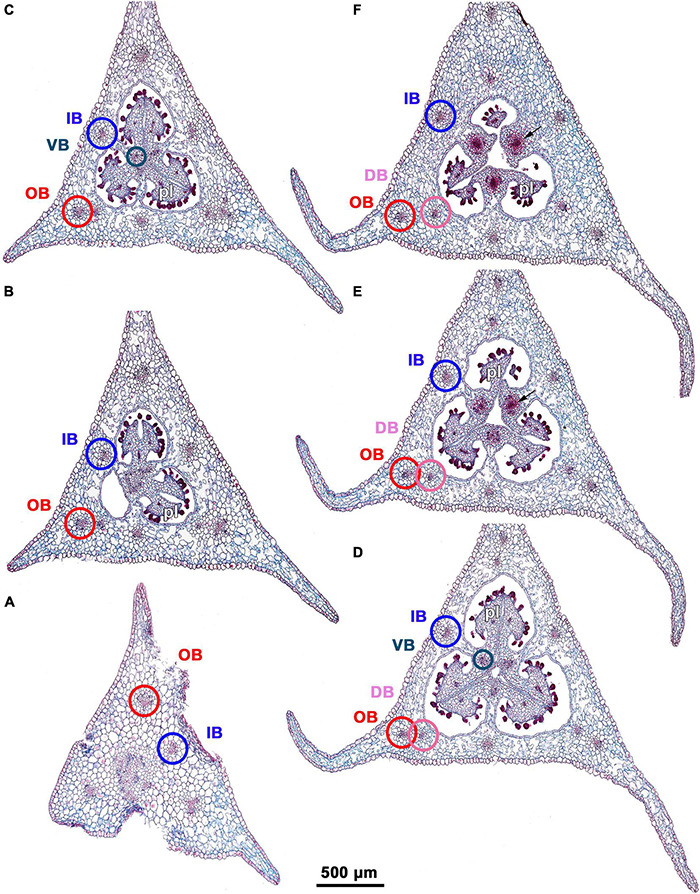
Ascending series of transverse microtome sections of flower bud of *Burmannia disticha* (LM). **(A)** Pedicel just below ovary. **(B)** Proximal (secondarily trilocular) portion of symplicate zone of ovary. **(C)** Middle (secondarily trilocular) portion of symplicate zone of ovary. **(D–F)** Distal (unilocular) portion of symplicate zone of ovary. Arrow indicates septal nectary. pl, placenta; DB, dorsal bundle; IB, inner bundle; OB, outer bundle; VB, ventral bundle.

**FIGURE 4:**
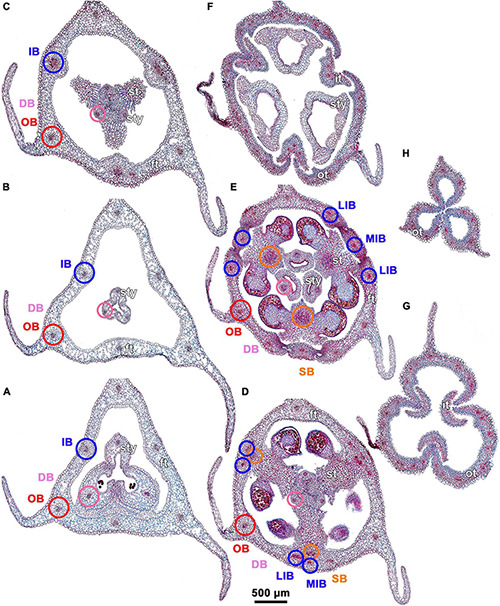
Ascending series of transverse microtome sections of flower bud of *Burmannia disticha* (continued from Figure 3). **(A)** Flower at transition between ovary roof and common style. **(B)** Flower at level of common style. **(C)** Flower at level of basal spurs of anthers. **(D)** Flower at level of gynostegium. **(E)** Flower at level of anthers and style branches. **(F)** Flower at level of stigmas and perianth lobes. **(G)** Outer and inner tepal lobes. **(H)** Outer tepal lobes. ft, floral tube; it, inner tepal; ot, outer tepal; st, stamen; sty, style; DB, dorsal bundle; IB, inner bundle; LIB, lateral inner bundle; MIB, median inner bundle; OB, outer bundle; SB, stamen bundle.

**FIGURE 5:**
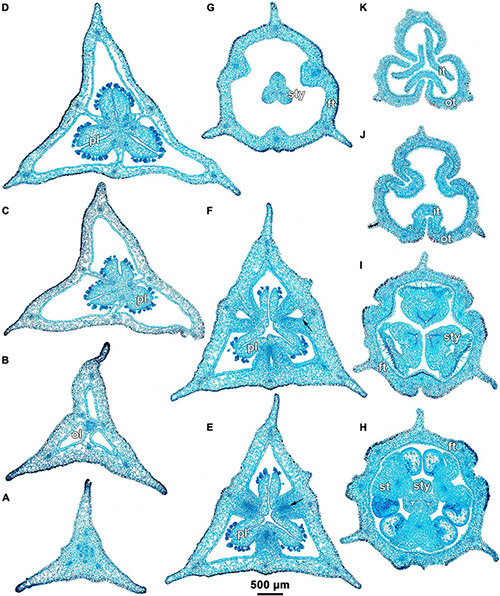
Ascending series of transverse microtome sections of flower bud of *Burmannia longifolia* (LM). **(A)** Pedicel just below ovary. **(B)** Synascidiate zone of ovary. **(C,D)** Secondarily trilocular portion of symplicate zone of ovary. **(E,F)** Distal (unilocular) portion of symplicate zone of ovary. **(G)** Flower at level of common style. **(H)** Flower at level of anthers and common style. **(I)** Flower at level of stigmas. **(J,K)** Outer and inner tepal lobes. Arrow indicates septal nectary. ft, floral tube; it, inner tepal; ol, ovary locule; ot, outer tepal; pl, placenta; st, stamen; sty, style.

**FIGURE 6:**
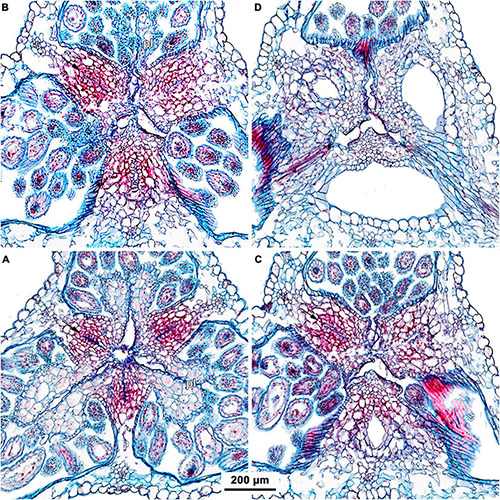
**(A–D)** Ascending series of transverse microtome sections of flower bud of *Burmannia coelestis* (*Nuraliev et al. 2736*) showing structure of septal nectaries (LM). Arrow indicates septal nectary. pl, placenta.

**FIGURE 7:**
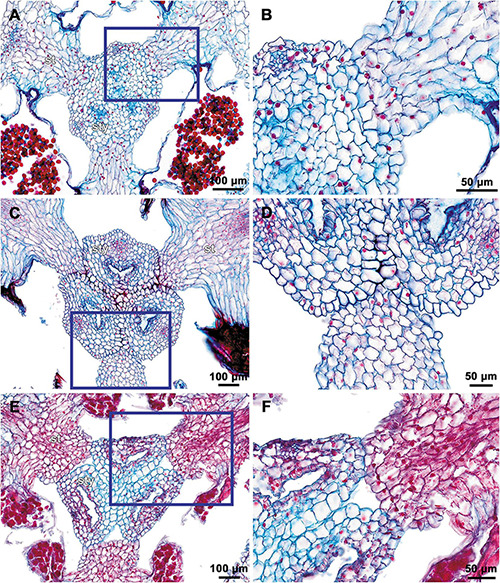
Transverse microtome sections of flower buds of *Burmannia* (LM) showing interactions between stamens and common style. **(A,B)** *B. oblonga* (*Poyarkov s.n.*), gynostegium with undetectable boundaries of fused stamens and common style (and epidermal cells along the line of fusion). **(C,D)** *B. itoana*, gynostegium with recognizable boundaries of fused stamens and common style (epidermal cells differ from surrounding cells). **(E,F)** *B. coelestis* (*Nuraliev et al. 2736*), stamens connivent with common style without fusion; central part of connective separated from common style by a gap. **(B,D,F)** Portions of sections contoured with blue in **(A,C,E)** respectively. st, stamen; sty, style.

**FIGURE 8:**
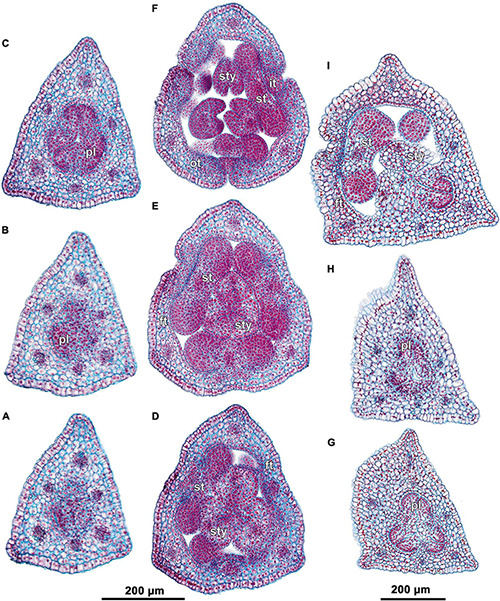
Transverse microtome sections of underdeveloped flowers of *Burmannia* (LM). **(A–F)** *B. lutescens* (*Nuraliev, Lyskov NUR 3120*). **(A)** Ovary base. **(B)** Synascidiate zone of ovary. **(C)** Symplicate zone of ovary. **(D,E)** Flower at level of anthers and common style (style fusion not yet completed). **(F)** Flower at level of perianth lobes. **(G–I)** *B. coelestis (Nuraliev et al. 2736*). **(G,H)** Symplicate zone of ovary. **(I)** Flower at level of bases of styles. ft, floral tube; it, inner tepal; ot, outer tepal; pl, placenta; st, stamen; sty, style.

For light microscopy (LM), at Moscow State University, cross sections of flowers were prepared using standard methods of Paraplast embedding and serial sectioning at 15 μm thickness (Barykina et al., 2004) using a Microm HM 355S automatic rotary microtome with HP35 Ultra disposable blades (Figures 3–8 and Supplementary Figures 1–6). The sections of *Burmannia championii* and *B. longifolia* were stained with alcian blue and mounted in DPX mounting medium; sections of other species were stained with alcian blue and safranin using a Shandon Varistain Gemini slide stainer and mounted in VitroGel mounting medium. Sections were examined using an Olympus BX53 light microscope equipped with an Olympus SC50 digital camera; images were also taken using Olympus Virtual Slide microscope VS120-S6-W.

**FIGURE 9:**
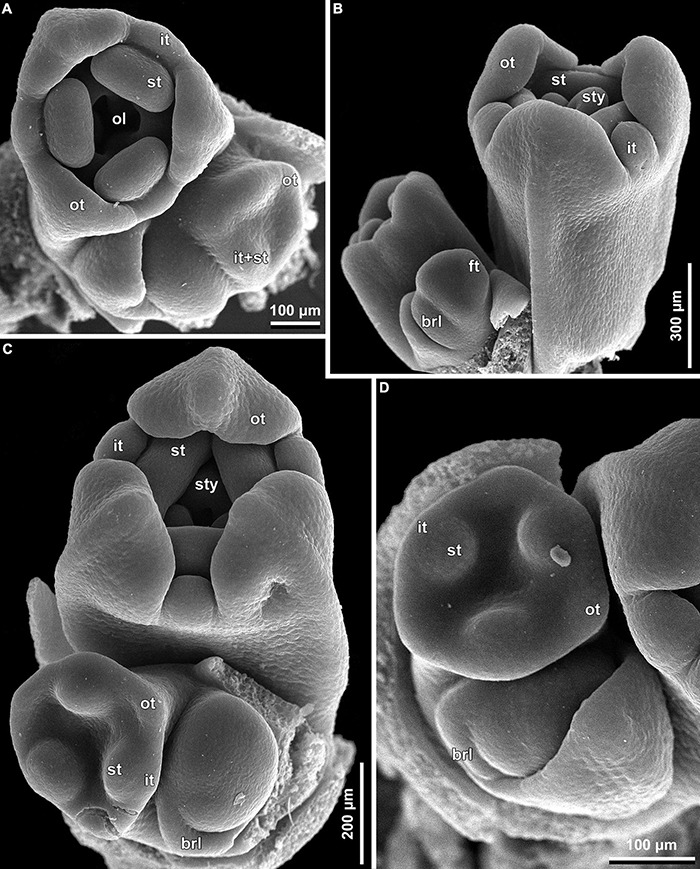
Early floral development of *Burmannia disticha* (SEM): portions of cincinni in top **(A,C,D)** and side **(B)** views. brl, bracteole; ft, floral tube; it, inner tepal; ol, ovary locule; ot, outer tepal; st, stamen; sty, style.

**FIGURE 10:**
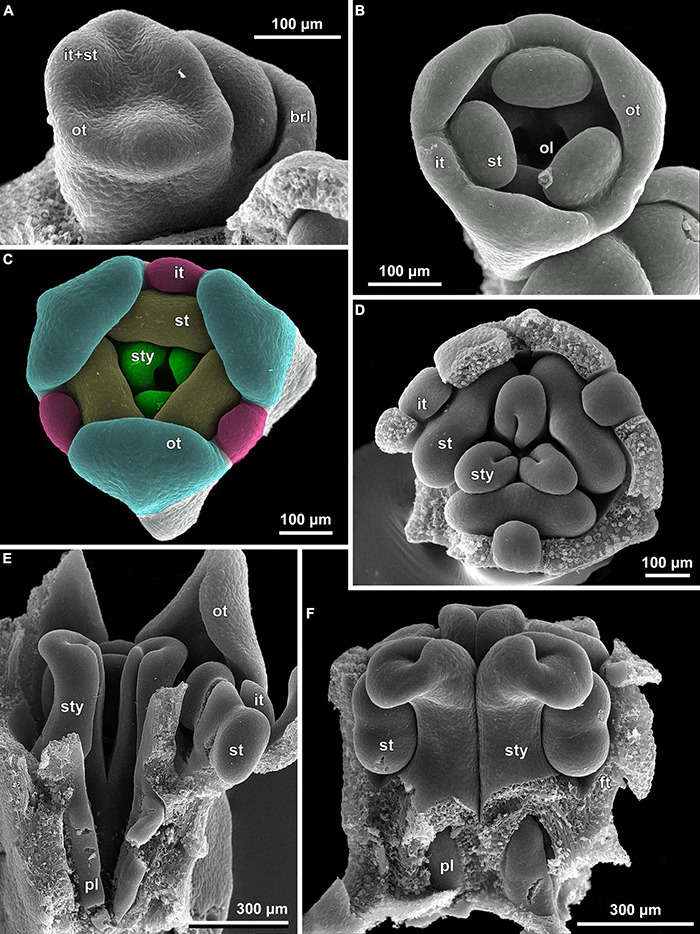
Floral development of *Burmannia disticha* (SEM). **(A)** Flower with primordia of outer tepal lobes and common primordia of inner tepal lobes and stamens. **(B)** Flower after initiation of ovary locule. **(C)** Flower after initiation of styles. **(D,E)** Flowers before congenital fusion of styles; top view with outer tepal lobes removed **(D)** and longitudinal section **(E)**. **(F)** Flower at congenital fusion of styles and development of placentas, longitudinal section. brl, bracteole; ft, floral tube; it, inner tepal; ol, ovary locule; ot, outer tepal; pl, placenta; st, stamen; sty, style.

**FIGURE 11:**
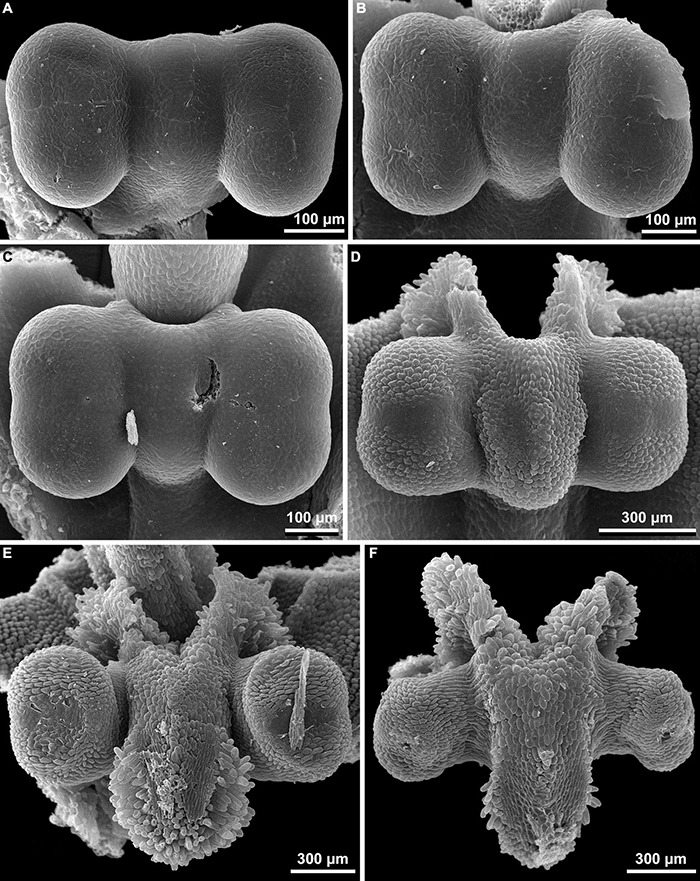
**(A–F)** Stamen development of *Burmannia disticha* (SEM).

**FIGURE 12:**
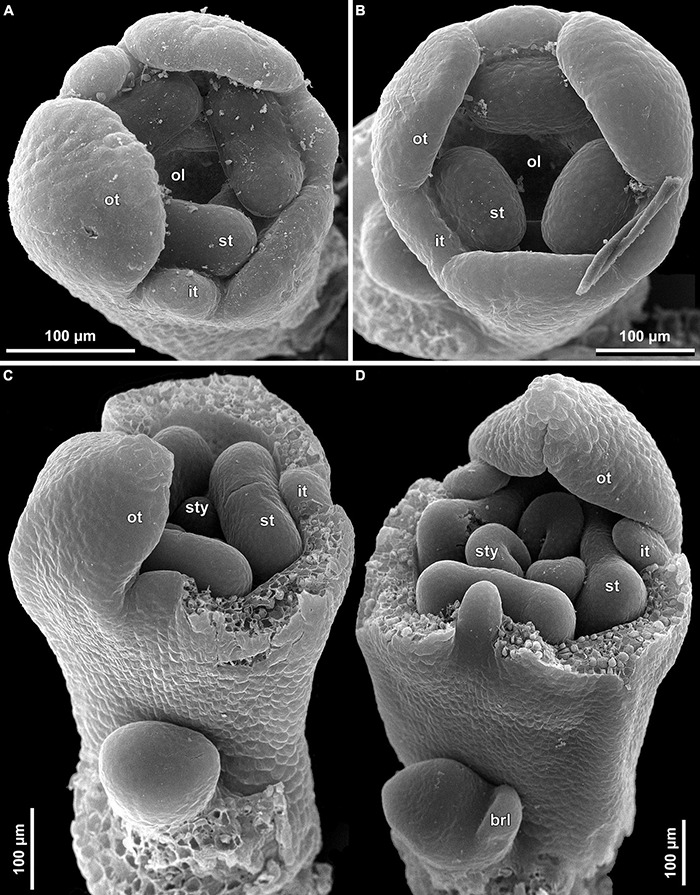
Floral development of *Burmannia* (SEM). **(A)** *Burmannia chinensis*. Flower after initiation of ovary locule; one outer tepal lobe is significantly larger than the others. **(B–D)** *Burmannia coelestis* (*Truong Ba Vuong, Dang Van Son BV1000*). **(B)** Flower at initiation of ovary locule. **(C,D)** Flowers at initiation **(C)** and development **(D)** of styles; two outer tepal lobes removed. brl, bracteole; it, inner tepal; ol, ovary locule; ot, outer tepal; st, stamen; sty, style.

**FIGURE 13:**
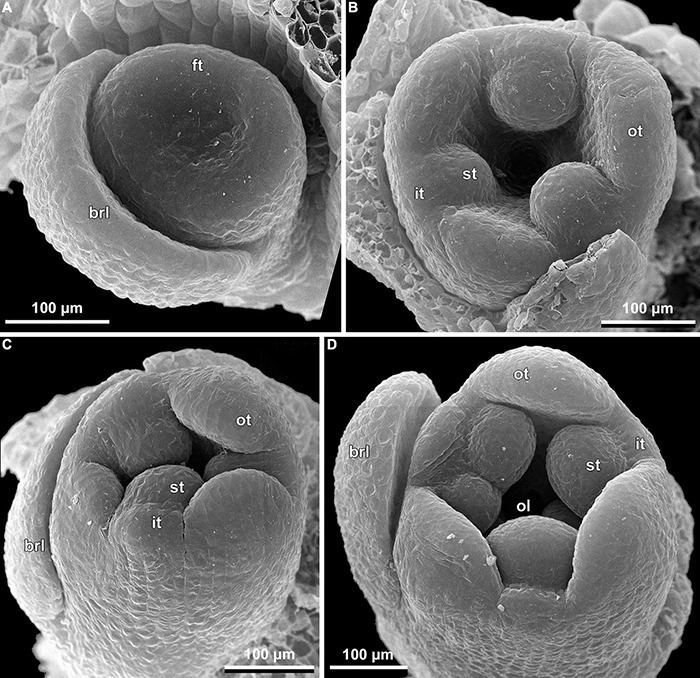
Early floral development of *Burmannia itoana* (SEM). **(A)** Flower at initiation of floral tube. **(B,C)** Flower at initiation of inner tepal lobes and stamens on their common primordia; in **(C)** one outer tepal lobe is smaller than the two others. **(D)** Flower at initiation of ovary locule; note the fourth (teratological) stamen. brl, bracteole; ft, floral tube; it, inner tepal; ol, ovary locule; ot, outer tepal; st, stamen.

**FIGURE 14:**
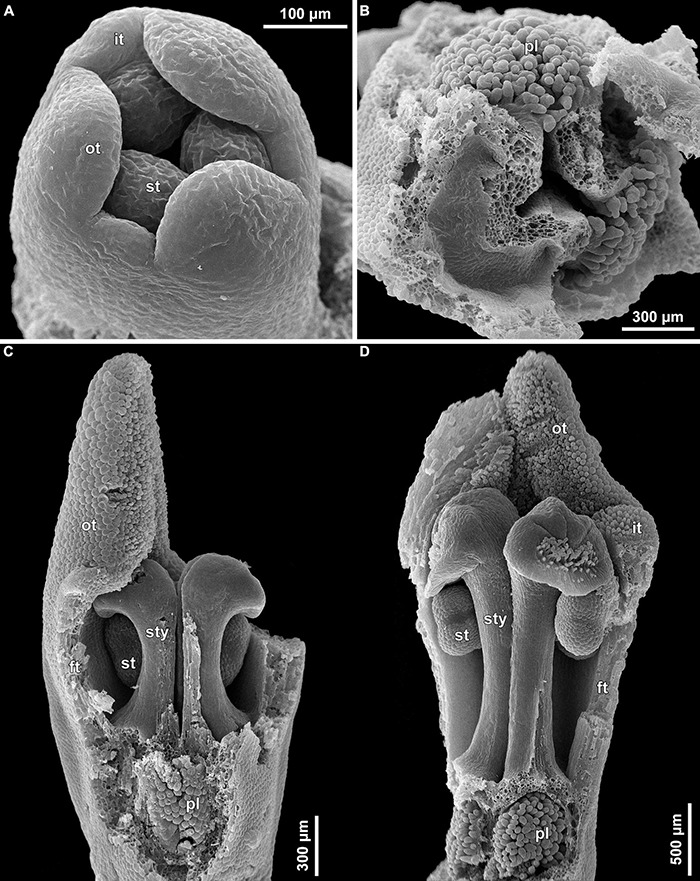
Floral development of *Burmannia itoana* (SEM). **(A)** Flower before initiation of gynoecium. **(B)** Ovary, top view; styles removed; ovary wall and roof in two of the three carpels removed. **(C,D)** Flowers before **(C)** and after **(D)** postgenital fusion of styles, longitudinal sections. ft, floral tube; it, inner tepal; ot, outer tepal; pl, placenta; st, stamen; sty, style.

**FIGURE 15:**
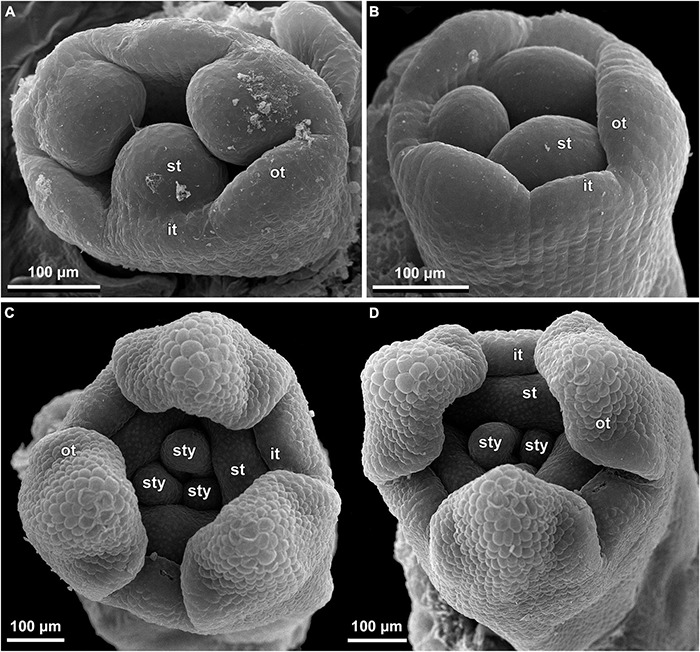
Floral development of *Burmannia oblonga* (SEM) (*Nuraliev, Lyskov NUR 3160*). **(A)** Flower at initiation of inner tepal lobes and stamens on their common primordia. **(B)** Flower at a stage slightly later than in **(A)**. **(C,D)** Flower at initiation of styles (one style shorter than the two others). it, inner tepal; ot, outer tepal; st, stamen; sty, style.

For scanning electron microscopy (SEM), material was dissected in 70% ethanol under an Olympus SZX7 stereomicroscope, dehydrated through 96% ethanol followed by absolute acetone, critical-point dried using a Hitachi HCP-2 critical point drier, then coated with gold and palladium using an Eiko IB-3 ion-coater. Observations were made using a CamScan S2 SEM at Moscow State University (Figures 9–15 and Supplementary Figure 7).

All images were processed using Adobe Photoshop Elements 2021. One SEM image was colored using CorelDRAW X5. In the Figures, each series of microtome sections is arranged in an acropetal manner, so that the most proximal section (labeled as A) occupies the bottom-left corner of the plate, and the most distal section occupies the top-right corner.

## Results

### Inflorescence Structure

The examined species of *Burmannia* are characterized by few- to several-flowered inflorescences. The above-ground shoot bears several bracts inserted following a 1/3 spiral, and ends in a terminal flower (Figures 1B–H). The outer tepals of the terminal flower alternate with the three uppermost bracts. The two uppermost bracts are placed close to the terminal flower, thus referred here to as involucral bracts; the other bracts (= stem leaves) are divided from them and from each other by long internodes. The involucral bracts are morphologically similar to the stem leaves, but in photosynthetic species they are usually smaller. The involucral bracts have either monochasial cymes (cincinni) or individual flowers in their axils. In addition, solitary flowers are usually present in axils of several stem leaves below the involucre; however, we never observed these flowers to be fully developed. In *B. disticha*, some stem leaves subtend severely underdeveloped cymes.

Several-flowered inflorescences were observed in *B. disticha* (with more than ten flowers, excluding those in axils of stem leaves; Figures 1B,E) and *B. lutescens* (with up to eight flowers; Figures 1B,F); inflorescence of these species is a thyrsoid (here and below, terminology after Endress, 2010). The involucral bracts have monochasial cymes in their axils. Within a monochasial cyme, each flower possesses a single floral prophyll (bracteole), which is arranged transversally or nearly transversally. A lateral flower of the next order developed in the axil of the floral prophyll thus has a transversal position. Along the cyme, there is an alternation of right and left positions of the floral prophylls, which leads to an apparently two-rowed arrangement of the flowers. The cyme with such a branching pattern corresponds to a cincinnus (Buys and Hilger, 2003). *Burmannia disticha* shows the longest cincinni: they often have more than six orders of branching, i.e., bear more than six flowers. In the first flower of the upper cyme, the floral prophyll is usually in an anodic position, i.e., closer to the end of the ontogenetic spiral (see Korn, 2006 for terminology). In the lower cyme, in contrast, the floral prophyll of the first flower is in a cathodic position. Only in some inflorescences of *B. lutescens*, the floral prophyll of the first flower is in anodic position in both cymes. In all lateral flowers, one of the inner tepals lies in the same radius with the floral prophyll; this allows compact packaging of the flowers, placing them side by side, with wings of the neighboring flowers more or less parallel to each other (Figure 1).

In few-flowered species, the inflorescences consist of up to four flowers (excluding flowers in axils of stem leaves) in *B. coelestis* (Figures 1C,G), up to three flowers in *B. itoana* and *B. oblonga*, up to two flowers in *B. chinensis* (Figures 1D,H). In all these species, the axils of the involucral bracts usually bear solitary flowers; in *B. chinensis* and *B. itoana*, only the lower involucral bract subtends a lateral flower, whereas the upper one lacks flowers in its axil. Such an inflorescence can be described as a botryoid; it is structurally similar to the thyrsoid found in the several-flowered species, differing in the presence of single lateral flowers instead of cincinni. In some individuals of *B. coelestis*, and rarely also in the other species, flowers of an additional order were observed, with the inflorescences approaching a thyrsoid. The arrangement of the floral prophylls in the few-flowered species, except for *B. oblonga*, is the same as in the several-flowered species. In *B. oblonga*, the floral prophylls are nearly adaxial median (at a slight angle to a strictly median position). The flower is oriented in such a way that one of its inner tepals occupies the radius of the floral prophyll, i.e., uniformly with the other species with respect to the floral prophyll, but differently from the other species with respect to the flower-subtending bract. In addition, *B. oblonga* differs from the other species studied in the involucral bracts inserted on stem slightly below the ovary base (with the flower thus being evidently pedicellate), and in a subopposite arrangement of the involucral bracts.

### Floral Structure

#### Floral Tube and Perianth

The species of *Burmannia* studied here possess tetracyclic trimerous bisexual flowers with long floral tube (Figures 1A, 2). The floral tube has variously shaped perianth wings in the radii of the outer tepals. The tepal lobes are placed at the top of the floral tube. Three stamens are inserted on the inner surface of the floral tube close to its top; the stamens occupy the radii of the inner tepals. In most of the species (e.g., *B. chinensis*, *B. coelestis*, *B. itoana*, *B. lutescens*, *B. oblonga*), the stamens are placed distinctly below the upper edge of the floral tube, i.e., there is a portion of the tube above the level of stamen insertion.

Interspecific differences in morphology of floral tube, tepal lobes and stamens are presented in Table 2. *Burmannia oblonga* is the only species examined which is characterized by the absence of the inner tepal lobes. The inner tepal lobes are present in both specimens of *B. lutescens*.

**TABLE 2:** Gross morphology of perianth and androecium in the studied specimens of *Burmannia*.

Species	Shape of perianth wings	Outer tepal lobes: shape and surface	Inner tepal lobes: presence, shape and surface	Shape of stamens
*B. championii*		Adaxially papillose, single-margined	Present, adaxially papillose, single-margined	
*B. chinensis*	Semi-elliptical or broadly semi-obovate, swirling around the floral tube	More or less triangular, adaxially papillose in basal half, deeply double-margined along the entire length, erect	Present, lanceolate, adaxially smooth, single-margined	High apical crests, long acuminate basal spur
*B. coelestis*	Semi-obovate	More or less triangular, adaxially smooth, deeply double-margined along the entire length, erect	Present, lanceolate, adaxially smooth, deeply double-margined	High apical crests, short acuminate basal spur
*B. disticha*	Semi-elliptical	More or less triangular, adaxially papillose, slightly double-margined at base, erect	Present, lanceolate, adaxially papillose, slightly double-margined at base	High apical crests, long obtuse basal spur
*B. itoana*	Semi-cuneate	Triangular, thickly papillose on the entire adaxial side, single-margined, erect	Present, semi-orbicular, adaxially thickly papillose, single-margined	Low apical crests, short acuminate basal spur
*B. longifolia*		Adaxially papillose, slightly double-margined at base	Present, adaxially papillose, single-margined	
*B. lutescens*	Semi-elliptical or narrowly semi-elliptical	Ovate, thickly papillose on the entire adaxial side, minutely double-margined at base, twisted on the abaxial side	Present, semi-orbicular to shortly rectangular, adaxially thickly papillose, single-margined	Low apical crests, basal spur lacking
*B. oblonga*	Semi-quadrangular	Nearly quadrangular, emarginate, thickly papillose on the entire adaxial side, single-margined, erect	Absent	Low apical crests, basal spur lacking

*For B. championii and B. longifolia, some of the characters are missing because SEM investigations were not performed.*

#### Androecium

Stamens are sessile or nearly sessile. The connective is broad, obovoid in front view. The connective has an elaborate structure: it is usually provided with two apical appendages (apical crests) and a basal appendage (basal spur), which are covered by numerous papillae, and with a central longitudinal rib (see Table 2). Each theca is nearly globose; it consists of two microsporangia arranged one above the other and divided by a horizontal septum (i.e., the microsporangia are superposed). The theca dehisces latrorsely by a transverse slit.

#### Gynoecium

The three carpels are congenitally united up to the ovary top, forming a syncarpous gynoecium with inferior ovary. In the superior part of the gynoecium, the carpels are initially free. Thus, the ovary roof and the styles represent an asymplicate zone. The inferior ovary is trilocular from its inception proximally (synascidiate zone) and secondarily trilocular distally (symplicate zone). Along the most symplicate zone, the lines of postgenital closure of ventral slits are undetectable in the cross section, i.e., the postgenital fusion is of perfect type (sensu Sokoloff et al., 2018). In the distal part of the symplicate zone of *B. disticha* (Figures 3E,F), *B. itoana* (Supplementary Figure 4E) and *B. longifolia* (Figure 5E), the postgenital closure of ventral slits (i.e., fusion of the septae) does not occur, and this part of ovary is unilocular. The placentation is axile (and parietal in the unilocular part of the ovary, when present). The placentas are Y-shaped in longitudinal view, as they are solid in the synascidiate zone and bilobed (following their arrangement along the carpel margins) in the symplicate zone. The solid portion of the placenta is sometimes basally not attached to the wall of the ovary locule, hanging into the upper part of the synascidiate zone (Supplementary Figure 4C). The placentas are intrusive; they are covered with numerous ovules over their entire surface. The small supralocular septal nectaries are located above the level of ovule attachment just below the ovary roof (Figures 3E,F, 5F, 6 and Supplementary Figures 3F, 4F). The nectaries open via three slits at the style bases. In the synascidiate and symplicate zones of *B. chinensis* and *B. coelestis*, the tissue in the gynoecium center is lignified to form a central column (Supplementary Figures 2A–D, 3A–E). In *B. lutescens*, tissue of this type is found only at the base of the gynoecium (Supplementary Figures 5A,B). In *B. championii*, *B. disticha*, *B. itoana*, *B. longifloia*, and *B. oblonga*, a lignified central column was not observed. The styles are postgenitally fused by their lateral sides (i.e., marginal areas of the abaxial surfaces) into a long filiform hollow common style. The lines of postgenital fusion between the styles are hardly recognizable in the cross sections, indicating perfect postgenital fusion. The most distal carpel parts are free and represent the three style branches, located above the stamens. Each style branch possesses a well-discernible ventral slit; the style branch contains a canal inside which continues downwards into the canal of the common style, and opens apically into the stigma. The canal of the common style is usually triradiate in the cross section, because the ventral slits remain open. The stigmas are broad, bent at ca. 90° to the abaxial side so that they face the perianth, each bearing a transverse folding covered by numerous papillae. The style branches and stigmas almost completely obstruct the orifice of the floral tube.

#### Interactions Between Androecium and Gynoecium

The stamens are placed just below the stigmas. In *B. chinensis* (Supplementary Figure 2G) and *B. oblonga* (Figures 7A,B), the ventral surfaces of the connectives contact the apical part of the common style and are postgenitally fused to the common style forming a gynostegium. The cells along the lines of postgenital fusion viewed in the cross sections have the same shape as the other cells of the corresponding organs, suggesting re-differentiation of the epidermis. The contact between the stamen and style cells does not differ from the intercellular contacts within an organ. In *B. disticha* (Figure 4D), *B. itoana* (Figures 7C,D), and *B. lutescens* (Supplementary Figure 5F), the connectives are also postgenitally fused to the common style to form a gynostegium, but the contact is apparently not as tight as in the previous case. Here, the elongated epidermal cells (in the plane of cross section) are distinguishable along the lines of fusion, and narrow gaps are visible between some of the contacting cells (Figures 7C,D). In *B. championii* (Supplementary Figure 1H), *B. coelestis* (Figures 7E,F) and *B. longifolia* (Figure 5H), connectives touch the common style by their edges, but the central ribs of the connectives do not reach the grooves between the carpels.

The thecae of each stamen are arranged close to the lower surfaces of the two neighboring stigmas, and the apical crests of the connective project to the gap between the two stigmas. Thus, the stamen-style complex of all the studied species (irrespective of the presence of fusion between the stamens and the common style) contributes to formation of a closed floral chamber, which cannot be reached from the outside without mechanical shifting of some of these organs.

### Floral Vasculature

Floral vasculature of eight species studied here shows the same groundplan. Below we provide a detailed description of the floral vasculature in *B. disticha* (Figures 3, 4). It has the most extensive vascular system of all species examined. This is followed by a description of the recognized interspecific differences.

Seven vascular bundles enter the receptacle from the pedicel (Figure 3A). Of them, one bundle occupies the central position (referred here to as a central bundle), and six bundles are arranged in two alternating rings with three bundles in each ring lying in the radii of corresponding tepals. The bundles of the outer ring occur in the radii of the outer tepals (and also the perianth wings and the styles); they are referred here to as outer bundles (OBs). The bundles of the inner ring occupy the radii of the inner tepals (and also the stamens and the ovary septae); they are referred here to as inner bundles (IBs).

The central bundle enters the ovary base. At the level of the bases of placentas in the synascidiate zone, the central bundle divides into three branches which become the three synventral bundles, located in the radii of septae (i.e., occupy intercarpellary, = heterocarpellary, positions) (Figure 3B). Each synventral bundle gives off two centrifugal branches (placental bundles). Each branch innervates a corresponding part of the placenta and runs along margins of two neighboring carpels (Figures 3C,D). The ventral bundles (three synventral and six placental ones) reach the ovary roof where they terminate (Figure 3F).

Within the ovary wall, each OB gives off a centripetal radial branch (dorsal carpellary bundle) (Figure 3D), which passes through the ovary roof (Figure 4A) and innervates the style (Figures 4B–E). The IBs continue directly into the floral tube. Ultimately, the floral tube receives six bundles: three OBs alternating with three IBs (Figure 4B).

Below the level of stamen attachment, each IB gives off three branches: two tangential bundles (referred to as lateral inner bundles, one at each side of the median inner bundle) and one centripetal radial bundle (stamen bundle) (Figures 4C,D). Each stamen bundle enters the stamen which occupies the same radius (Figure 4E). Thus, above the level of stamen attachment the floral tube contains 12 bundles, of which three groups of inner bundles (one median and two lateral ones in each group) alternate with three OBs. All of them run into the tepal lobes (Figure 4E).

Each outer tepal lobe receives an OB, which becomes its median trace, and one lateral inner bundle at each side (lateral traces). The inner tepal lobes are single-traced; each of them receives a median inner bundle (Figure 4E). In both outer and inner tepal lobes, branching of bundles takes place. In the outer tepal lobes, each lateral bundle gives off two tangential branches, resulting in seven bundles per tepal. The bundles of the inner tepal lobes trifurcate tangentially (Figures 4F,G).

*Burmannia longifolia* differs from *B. disticha* only in having unbranched bundles of the inner tepal lobes (Figure 5J).

*Burmannia championii*, *B. chinensis* and *B. coelestis* show identical floral vasculature. They differ from *B. disticha* in having unbranched bundles of both outer and inner tepal lobes (Supplementary Figures 1–3).

In *B. lutescens*, the median inner bundles terminate near the bases of inner tepal lobes; the inner tepal lobes are thus unvascularized. Bundles of the outer tepal lobes are unbranched (Supplementary Figures 5G,H).

In *B. itoana*, the differences from *B. disticha* are found in the innervation of the upper part of the floral tube and the perianth. The inner bundles give off only the stamen bundles within the floral tube (Supplementary Figure 4H), and end blindly below the bases of the inner tepal lobes. Thus, *B. itoana* has single-traced outer tepal lobes and unvascularized inner tepal lobes. The bundles of the outer tepal lobes are unbranched (Supplementary Figures 4J,K).

*Burmannia oblonga*, which is characterized by the absence of the inner tepal lobes, differs from *B. disticha* in the vascular system of the gynoecium and the upper part of the floral tube. First, the receptacle receives only the six peripheral bundles from the pedicel, whereas the central bundle is lacking (Supplementary Figure 6A). Each IB gives off two centripetal radial branches. At the bottom of the ovary, these branches merge pairwise forming three ventral bundles in the radii of the ovary locules (i.e., in homocarpellary positions) (Supplementary Figures 6B,C). Toward the upper part of the ovary, each ventral bundle bifurcates into placenta bundles (without prolongation beyond the point of branching). Second, below the level of stamen attachment, each IB gives off two branches in tangential direction (i.e., the lateral inner bundles) and continues into the stamen which occupies the same radius (Supplementary Figures 6F,G). Each pair of the lateral inner bundles continues into the two neighboring (outer) tepal lobes, and the OBs become the median traces of the tepal lobes, just as in *B. disticha* (Supplementary Figure 6H). The bundles of the tepal lobes are unbranched.

### Floral Development

The floral development of the six species of *Burmannia* studied here is essentially uniform. Below we provide a detailed description for *B. disticha*, as we obtained the most complete data for this species. We then describe the additional observations made for the other species.

(1) The terminal flower is initiated at the apex of the above-ground shoot. To produce the terminal flower, the shoot apical meristem becomes converted into a floral one. The lateral flowers are initiated in the axils of flower-subtending bracts. For the first flower of a cyme, the flower-subtending bract is an involucral bract below the terminal flower. For all the subsequent flowers of a cyme (if any), the flower-subtending bract is a floral prophyll on the pedicel of the preceding flower (Figure 9A: the young flower with a lateral primordium). Floral primordia are slightly compressed in a transverse direction (because of pressure of the large subtending bract). Then, the floral primordium expands laterally and the floral prophyll appears in an (almost) transverse position. Almost simultaneously with the initiation of the floral prophyll, a flower of the next order becomes visible in the axil of the floral prophyll (Figures 9B,C).
(2) When the flower reaches c. 250 μm in diameter, the receptacle becomes concave, which can be interpreted as a first sign of appearance of the floral tube in the form of a low ring-like primordium (Figure 9B: the youngest flower). Soon after that, six primordia become visible as low bulges on the top surface of the floral tube (Figure 9A: right flower, Figure 10A). Three of them are the primordia of outer tepal lobes. They are nearly twice smaller than three larger primordia. The larger primordia are the common primordia of inner tepal lobes and stamens. The outer tepal lobes are obtusely triangular, whereas the inner primordia are more roundish at this stage.
(3) When the flower reaches c. 350 μm in diameter, the common inner (tepal-stamen) primordia elongate radially and soon divide by a shallow constriction into tepal and stamen parts. At this stage, the floral apex becomes pronouncedly concave, which can be interpreted as formation of the ovary locule (i.e., symplicate zone of the gynoecium) (Figure 9C: left flower, Figure 9D: older flower).
(4) When the flower reaches c. 450 μm in diameter, the styles develop as plicate structures alternating with stamens. The styles are horseshoe-shaped in top view, showing broadly open ventral furrow. The ovary locule is clearly recognizable as a prominent depression between the styles. At this stage, the stamens increase in size and acquire oval roller-like shape. Outer tepal lobes expand laterally and overlap the stamens, and acquire abaxial median ribs (Figure 9A: the oldest flower, Figure 10B).
(5) Further, the flower elongates significantly, reaching c. 850 μm in height, while its diameter remains close to 450 μm (Figures 9B: the oldest flower, Figure 10C). The elongation occurs as a result of ovary growth, whereas the floral tube remains very short and hardly recognizable at this stage. Stamens expand further tangentially, almost touching each other at their lateral margins. Each stamen differentiates into a wide connective and two nearly globose thecae. At this stage, the styles begin to elongate but are still shorter than the stamens. From this stage, the outer tepal lobes are several times larger than the inner ones.
(6) When the flower reaches c. 600 μm in diameter, the styles elongate and overtop the stamens. Their apices bend outwards, indicating the first sign of stigma formation (Figure 10D). The symplicate zone of the gynoecium continues to elongate (Figure 10E). The outer tepal lobes are triangular in outlines, boat-shaped, with margins overlapping the inner tepal lobes. The inner tepal lobes are rectangular at this stage and slightly inclined toward the floral centre, touching the stamens (Figure 10E). Soon after that, parietal placentas appear as protrusions into the ovary locule in the symplicate zone (Figure 10F), with two placenta lobes on each ovary septa in the radius of the inner tepal.
(7) At late stages, the floral tube elongates dramatically, eventually becoming longer than any other part of flower. Stamens become papillose and tightly appressed to the styles under the stigmas. Two apical crests running along the dorsal side of the stamen, and a basal spur differentiate in the connective (Figures 11B–D). These structures soon become covered with hairs (Figures 11D–F). Free carpel parts (comprising the asymplicate zone) fuse postgenitally to form the common style and the ovary roof, with a central canal left. This fusion event also results in formation of supralocular septal nectaries. The stigmas become broader than long with their apices folded on the dorsal side, and a ventral seam at the center on the upper part. The synascidiate zone of the gynoecium arises. Placentas become Y-shaped (in longitudinal view) and intrusive. Numerous ovules initiate on the placentas occupying their entire surface. Finally, the ventral slits in the symplicate zone close postgenitally resulting in trilocular ovary, except for its distal part that remains unilocular.

In early development of perianth of *B. chinensis* (Figure 12A), *B. coelestis* (Figure 12B), and *B. itoana* (Figures 13B–D), the outer tepal lobes are often prominently unequal in size. This asymmetry seems not to be related to the position of the flower-subtending bract, although our material does not allow to verify it in details. Apparently, the asymmetry disappears at middle or late stages. In *B. itoana*, the stage of the drastic elongation of the floral tube (synchronized with elongation of the common style) was recognized to correspond to the diameter of flower between 1.3 and 1.8 mm (Figures 14C,D), i.e., far after all the main floral parts are completely formed.

Floral development of *B. oblonga* was traced since the stage of initiation of the inner tepal lobes and stamens from their common primordia, when the flower is c. 300 μm in diameter (Figures 15A,B). At this stage, the outer tepal lobes alternate with slightly elevated sectors of the floral tube. We consider these elevations to represent the inner tepal lobes. They are evident at least till the outer tepal lobes begin to cover the flower (which becomes c. 700 μm in diameter), but completely vanish by the stage of a preanthetic flower.

For *B. coelestis* and *B. lutescens*, cross sections of flowers at early developmental stages were investigated (Figure 8). In a flower of *B. lutescens* c. 450 μm in diameter, a very short, apparently just initiated synascidiate zone of gynoecium was detected (Figure 8B). The symplicate zone is unilocular: the septae (bearing parietal placentas) are evidently free from each other (Figure 8C). In a flower of *B. coelestis* c. 700 μm in diameter, a synascidiate zone is still lacking. The ventral slits in the symplicate zone are already closed; the ovary is thus secondarily trilocular and the placentation is axile in this part. The developing flowers of *B. coelestis* are comparable in size with those of *B. disticha* at corresponding stages, being only slightly smaller (Figure 12B, 335 μm vs. Figure 10B, 350 μm; Figure 12C, 420 μm vs. Figure 10C, 500 μm; Figure 12D, 515 μm vs. Figure 10D, 650 μm). The same is true for *B. chinensis* (Figure 12A). This allows to assume the flowers of the same size to represent similar developmental stages in *Burmannia*.

Development of ovules was studied in *B. lutescens* (Supplementary Figure 7). In the symplicate zone, the ovules cover the entire surface of the two placenta lobes except for their sides that face each other (i.e., along the boundary between the neighboring carpels). The ovules are anatropous, bitegmic; the inner integument initiates before the outer one. The ovules are nearly sessile at early stages, and later a long funiculus develops.

## Discussion

### Inflorescences

The inflorescences of *Burmannia* with more or less numerous flowers are thyrsoids with two lateral monochasial cymes. The botryoids of the few-flowered species possibly evolved from the thyrsoids as a result of reduction of monochasia; this idea is consistent with the available phylogenetic data (Merckx et al., 2008; Zhao et al., 2021). The presence of floral prophylls, their number, position and thus the inflorescence type are usually conserved at the genus or family level in monocots (Remizowa et al., 2013). The occurrence of a floral prophyll provides the possibility of further branching in its axil and leads to formation of a monochasium (instead of a solitary flower in the axil of a flower-subtending bract on the primary inflorescence axis). Thus, in taxa with floral prophylls present, the racemose inflorescences are structurally close to thyrses (see also Nuraliev et al., 2016, 2020).

An evolutionary transition from a raceme to a thyrse (and vice versa) is possible, but the presence of prophylls on the floral pedicels represents a key condition for such a transition. All members of Dioscoreales constantly develop floral prophylls, but their number and position are variable. For example, in Dioscoreaceae and Nartheciaceae, the single floral prophyll is in transversal or nearly transversal position. Among Nartheciaceae, *Aletris* L., *Metanarthecium* Maxim. and *Narthecium* Huds. possess racemes, whereas *Lophiola* Ker Gawl. and *Nietneria* Benth. are characterized by thyrses with monochasia (Remizowa et al., 2006a,^2008^, ^2013^; see also Tobe et al., 2018). In genera with racemes, the floral prophyll is inserted either at the right or left side of the flower within the same inflorescence. In species with thyrses, the position of the floral prophyll is more precise. In *Dioscorea* L. (Dioscoreaceae), the female flowers are often in spikes and the male flowers are arranged in thyrses (Remizowa et al., 2010a). The branched male inflorescences of *Dioscorea* demonstrate alternation of right and left floral prophylls, which results in development of cincinni. The inflorescences of *Thismia* Griff. (Thismiaceae) are thyrsoids. *Thismia* is unusual in the variable number of floral prophylls (2 or 3) and preferred branching positions (Nuraliev et al., 2021). In *T. annamensis* K. Larsen and Aver. with three floral prophylls, the branching occurs in the axil of the abaxial median prophyll, and the monochasium is therefore a drepanium. In other species of *Thismia*, branching occurs in transverse plane in the axil of a lateral prophyll, and the inflorescence is a bostryx.

Species of *Burmannia* examined here constantly produce a single floral prophyll, but differ by its position and intensity of branching. Most of the species examined constantly produce transversal floral prophyll in a very precise position, resulting in formation of cincinni in species with several orders of branching. *Burmannia oblonga* is the only species with a nearly median (adaxial) floral prophyll. If there were flowers in the axil of this floral prophyll, this would lead to formation of another type of monochasial cyme: a rhipidium. As in the other monocots with a single floral prophyll, the flower orientation is dependent on position of the floral prophyll in *Burmannia*. The floral prophyll is inserted between the two outer tepals and in the same radius with an inner tepal.

### Floral Vasculature

The floral vasculature is remarkably uniform in all the species of *Burmannia* studied to date (Pai, 1966; Rao, 1969; Rübsamen, 1986, this study). Certain variation is found in the vascular system at the flower base. Pai (1966) reported six central bundles at base (instead of a single bundle found in most of the species), which fuse with each other pairwise to form the synventral gynoecium bundles. The synventral bundles are common for all the species except for *B. oblonga*; they are thus formed in different ways. In *B. oblonga*, there are no central bundles at the ovary base, and the ventral bundles appear as branches of the peripheral bundles. Most important, the three ventral bundles of *B. oblonga* occupy the positions in the radii of the ovary locules, in contrast to the positions of the synventral bundles (alternating with the locules) of the other species.

The vasculature of the perianth and the distal part of the floral tube is most diverse. The outer bundles of the floral tube uniformly supply the outer tepal lobes forming their median traces. The inner bundles in most of the species continue up to the inner tepal lobes (forming their median traces) and give the lateral branches that form lateral traces of the outer tepal lobes. However, in *B. oblonga*, the inner bundles do not project beyond the point of departure of the stamen bundles, which is consistent with the absence of the inner tepal lobes in this species. In *B. itoana*, the inner bundles do not send the lateral branches, and therefore the outer tepal lobes receive no lateral traces. Then, in *B. itoana* and *B. lutescens*, the inner bundles do not reach the bases of inner tepals, which does not affect the groundplan of the floral vascular system (see “Perianth: inner tepal lobes”), but makes these tepals unvascularized. Finally, the species of *Burmannia* differ from each other in the degree of branching of the bundles within the tepal lobes.

As follows from the drawings provided by Rao (1969), he has observed neither lateral traces of the outer tepal lobes nor lateral inner bundles in the floral tube in *B. disticha*. At the same time, the lateral bundles of outer tepal lobes are drawn by Rao (1969) for *B. nepalensis* and *B. pusilla*. To date, single-traced outer tepal lobes are known only in *B. itoana* studied here. Given the rarity of this feature in *Burmannia* and the three-traced nature of the outer tepal lobes in our specimen of *B. disticha*, we suppose that Rao overlooked these bundles in the latter species.

### Floral Tube: Issues of Terminology

A tube in a flower that bears all the floral elements except for the gynoecium fits the idea of a hypanthium (Leins and Erbar, 2010; Ronse De Craene, 2010). In most cases, it is impossible to prove the axial or appendicular nature of such a tube, and for this reason the morphological nature is currently not taken into account in definition of a hypanthium (Sokoloff et al., 2018). In *Burmannia*, the stamens are usually attached distinctly below the apex of the floral tube, and therefore only a part of the floral tube corresponds to the hypanthium (i.e., from the tube base to the level of stamen attachment). The rest of the floral tube (above the level of stamen attachment) bears only the free tepal lobes, and thus corresponds to the perianth tube. The perianth tube in *Burmannia* was overlooked by Caddick et al. (2000) who considered the entire floral tube to be formed by a hypanthium.

In this paper, we use the term “floral tube” for clarity, assuming that it consists of a hypanthium and a perianth tube, the latter being vanishingly short in some species of *Burmannia*. We have not observed any additional structural features that delimit hypanthium and perianth tube, apart from the level of stamen attachment and the pattern of branching of the vascular bundles related to the departure of the stamen bundles. Accordingly, the so-called perianth ribs or wings in *Burmannia* are morphologically parts of the ovary wall at their bases, hypanthium in the middle and perianth tube at the apex. There is no reason to consider the wings as expansions of the outer tepals, as was suggested by Pai (1966).

### Perianth: Tepal Margins

Both outer and inner tepal lobes in *Burmannia* are characterized by either single or double margins. Although these characters are widely used for species delimitation and identification in the genus (Jonker, 1948; Zhang, 1999; Wu et al., 2010), they have never been described for some of the species. We here supply the description of the species under study with information on tepal margins based on careful analysis of spirit material, SEM investigations and the cross sections (see Table 2). In particular, we report the shape of tepal margins for the first time for *B. longifolia* (outer tepals slightly double-margined at base, inner tepals single-margined) and *B. lutescens* (outer tepals minutely double-margined at base, inner tepals single-margined).

In tepals with double margins, the border between the adaxial and abaxial sides is not obvious. As follows from our observations on *B. chinensis* and *B. coelestis* (not shown), the double nature is a result of development of a concavity in a thick tepal margin. Thus, the surface of the double margin belongs neither to the adaxial nor to the abaxial side of tepal.

### Perianth: Inner Tepal Lobes

The most intriguing feature of perianth in *Burmannia* is the apparent absence of the inner tepal lobes in some cases. Some species, including *B. cryptopetala* Makino and *B. oblonga*, are uniformly characterized by this feature, whereas in other cases this feature contributes to uncertainties in species delimitation. The most prominent example is the *B. lutescens* species complex, which was divided into two species by Zhang (1999): the widely distributed *B. lutescens* (including *B. tridentata* Becc.), polymorphic with respect to this and several other characters, and *B. gracilis* Ridl. endemic to the Malay Peninsula with the inner tepal lobes present. Jonker (1938, 1948), in contrast, accepted *B. lutescens* (including *B. gracilis*) as uniformly possessing the inner tepal lobes, and *B. tridentata* as a distinct species known from a single collection made in Sarawak that lacks the inner tepal lobes. Although the former view, already adopted by Govaerts et al. (2011), is followed here, we argue that the taxonomy of this species complex is far from being resolved, and reliable morphological data is wanted, including the results of developmental studies and the information on structural variation in various geographical areas.

In *B. oblonga*, we observed the inner tepal lobes at the middle stages of floral development. They were found to be extremely short, but quite broad, being only twice narrower than the outer tepal lobes. Preanthetic flowers are shown to lack any evidences of the inner tepal lobes, and there are no vascular bundles in the floral tube corresponding to bundles of the inner tepals.

The two Vietnamese specimens of *B. lutescens* studied here are geographically remote from the rest of the known distribution area of this species, representing the only documented extra-Malesian populations (Nuraliev et al., 2018). Anthetic flowers of both specimens show distinct inner tepal lobes, which are unvascularized, but the bundles that act as median traces of the inner tepal lobes in the other species of *Burmannia* (i.e., the median inner bundles of the perianth tube) terminate right at the bases of the inner tepal lobes. One can interpret the absence of vascularization as a trend toward reduction of the inner tepal lobes in *B. lutescens*; possibly, in some populations of this species they are short enough to be hardly distinct in mature flowers. The same vascular pattern is found in *B. itoana*, which has even shorter (semi-orbicular) inner tepal lobes. However, *B. itoana* has never been reported to lack the inner tepal lobes (Jonker, 1938; Ohashi et al., 2000; Wu et al., 2010; Tsukaya, 2016).

In total, our data indicate that the inner tepal lobes are present in all species of *Burmannia* at least developmentally. Since most of the species possess well-developed and vascularized inner tepal lobes, and the species with a seeming absence of the inner tepal lobes (in maturity) or their vascular bundles are nested deep within the phylogeny of *Burmannia* (Merckx et al., 2006, 2008; Zhao et al., 2021), we assume that the deviations from this common pattern are cases of evolutionary reduction in the genus. The variation of morphology of the inner tepal whorl in the genus is likely to be quantitative (continuous) rather than binary, with several intermediate variants already recognized. It remains unclear, whether a considerable variation of degree of this reduction occurs at infraspecific level in *Burmannia*, or the presence/absence of the inner tepal lobes in maturity (and their vascular supply) is a species-specific character.

A tendency to perianth reduction in *Burmannia* mirrors a situation in Thismiaceae, another family of Dioscoreales. In some lineages of *Thismia*, there is a tendency to reduction of the outer tepals which are much smaller than the inner ones and do not contribute to the flower protection. Species of *Thismia* possess a rather uniform floral vasculature, with most prominent variation observed in the innervation of tepals. In *T. mucronata* Nuraliev (Nuraliev et al., 2021) and *T. puberula* Nuraliev (Yudina et al., unpublished), the small but well-visible outer tepals are non-vascularized, whereas the outer tepals of other species of *Thismia* studied are uniformly three-traced.

Interestingly, the evidences of reduction in the outer tepal whorl do not affect the number of stamens in *Thismia*: flowers of *Thismia* uniformly possess six stamens. In the development, stamens of *Thismia* appear as protrusions at bases of tepal primordia after the carpels are initiated (Nuraliev et al., 2021). This pattern resembles late division of common tepal-stamen primordia. In contrast, *Burmannia* demonstrates reduction in both perianth and androecium. Surprisingly, irrespective of larger outer tepals, the outer stamens are lacking in *Burmannia.* This pattern of stamen whorl reduction is apparently due to initiation of inner tepals and stamens via common primordia (which was not necessarily the case for outer tepals and outer stamens in a putative ancestor of *Burmannia*). We speculate that organs developing via common primordia cannot be modified independently in the course of evolution as they are united in a solid module. Indeed, the inner tepals are always initiated in *Burmannia* (although not always well-detectable in mature flowers).

### Stamens

Caddick et al. (2000) assumed the family Burmanniaceae to be the only lineage of Dioscoreales with the inner stamen whorl present and the outer one lacking. Most of Dioscoreales are characterized by two-whorled androecium, and examples of the taxa with a single stamen whorl being the outer one are provided by Caddick et al. (2000). In fact, the androecium structure found in Burmanniaceae is also characteristic of *Oxygyne* Schltr., a member of Thismiaceae (Shepeleva et al., 2020).

In this study, the entire process of androecium initiation and development is documented. No traces of initiation of the outer stamens were recognized. This allows to state that the outer stamens are structurally completely absent in *Burmannia*. However, the orientation of the inner androecium whorl and the gynoecium in *Burmannia* corresponds to that of a typical pentacyclic monocot flower, i.e., the inner stamens are in the radii of the inner tepals, and the carpels in the radii of the outer tepals. It is likely that the outer stamens do not develop morphologically, but act as a source of positional information governing the orientation of the subsequent floral whorls. This hypothesis corresponds to the idea of ablasted organs (Stützel and Gansser, 1995; Choob, 2010).

### Gynoecium Zonation

The common style in *Burmannia* is a complex structure, consisting of three postgenitally fused styles (belonging to three carpels), as has already been shown by Zhang (1999) and Caddick et al. (2000); it thus represents the asymplicate zone of gynoecium. In some other Dioscoreales, such as Thismiaceae (Nuraliev et al., 2021), some Nartheciaceae (Remizowa et al., 2006b,^2008^; Tobe et al., 2018), Taccaceae (Igersheim et al., 2001) the columnar portion of the gynoecium above the ovary is formed by the distal part of the symplicate zone, and usually bears free styles at its apex. It can be called a stylar column. We find it appropriate to apply distinct terms for these morphologically different structures, despite some authors use “style” also for the common style (Maas et al., 1986; Maas-van de Kamer, 1998; Merckx et al., 2013).

The identification of the border between the synascidiate and symplicate zones of gynoecium (i.e., the cross zone) in mature flowers of *Burmannia* is complicated due to postgenial fusion accompanied by complete re-differentiation of contacting epidermises at the base of the plicate zone of each carpel. As a result, the lines of postgenital fusion are indiscernible. The symplicate zone is thus proximally very similar to the synascidiate zone, as it is trilocular without distinct lines of postgenital fusion in the ovary center (in cross section). We based on the following evidences to identify the zones. (1) Shape of placentas: we assume that in the synascidiate zone, there are no morphogenetic reasons for development of the bilobed placentas (in cross section). Therefore, placentas of this structure indicate symplicate zone, and the entire (rounded) placentas indicate the synascidiate zone. (2) Developmental data: at the stage of already elongated ovary with no postgenital fusion yet occurred (e.g., Figure 10E), most of the ovary length is unilocular, thus representing the symplicate zone.

We conclude that all the studied species of *Burmannia* have uniform structure of the ovary, comprising a long symplicate zone and a much shorter synascidiate zone. The placentas occupy the entire symplicate zone, where each placenta in the secondarily trilocular part has a bilobed shape with the lobes corresponding to the fused carpel margins of the same carpel, and in the unilocular part (if present) it has a form of two separate strands. In the synascidiate zone, the placentation takes place only in a short distal part; since the placentas have hanging portions below the level of their attachment, they are found in the cross sections of the synascidiate zone for some length further down.

Pai (1966) stated the entire trilocular portion of the ovary in *B. pusilla* to be “a result of the intruding placentae meeting in the center,” i.e., belonging to the symplicate zone. However, Figure 2 of his work clearly shows the basal part of the ovary with round (and not bilobed) placentas in the cross section, indicative of the synascidiate zone. Pai’s observation therefore fits well our general conclusion on the structure of the ovary in *Burmannia*.

The presence of the common style formed by postgenitally fused individual styles (i.e., the asymplicate zone right above the ovary) correlates with presence of septal nectaries. Septal nectaries of *Burmannia* are confined to the plicate carpel zone and located in the distal region of the ovary above the level of ovule insertion; they open via slits at the ovary roof. Similar supralocular septal nectaries are also reported for *Dioscorea* (Caddick et al., 2000). In Natheciaceae (*Aletris* and *Metanarthecium*), septal nectaries are more pronounced; they are interlocular, occupying almost the entire length of the plicate carpel zone along the ovary. The level of nectary orifices depends on ovary position. In *Aletris*, characterized by semi-inferior ovary, the opening slits are located at the ovary roof as in *Burmannia*. In *Metanarthecium*, the ovary is superior and nectaries open via special ducts at the ovary base (Remizowa et al., 2008; Tobe et al., 2018).

### Gynostegium

Our study revealed synorganization of the stamens and the gynoecium in *Burmannia* which involves diverse structural interactions. In some of the species, the interaction is limited to the tight arrangement of the stamens around the common style (without organ fusion), with the thecae placed just below the broad stigmas, and the apical crests of the connectives projecting between the stigmas. In the others, we observed the connectives to be postgenitally fused to the common style, with their central longitudinal ribs fitting the grooves between the carpels. In the latter case, the stamen-style complex corresponds to the idea of a gynostegium earlier considered to be unique for some lineages of Apocynaceae (Endress, 2006, 2016). In the species of *Burmannia* with gynostegia, the stamens can hardly be detached from the common style without disruption of tissues in at least a part of the area of contact (e.g., Figure 11E). The gynostegia of *Burmannia* vary in the degree of the fusion, which is manifested in evidently or hardly visible boundary between the fused organs in the cross sections.

The fusion between the stamens and the common style has been reported by Pai (1966) for *B. pusilla*, who stated that “the stamens are connected with the style by three broad bands of parenchymatous tissue.” Rao (1969) failed to find any such connection in *B. disticha* and *B. nepalensis*. It is difficult to compare their observations with our results, because no appropriate illustrations were provided by these authors.

### Reproduction

All the structures described above are ultimately involved in the process of seed production. The complex floral construction and the reward offered via septal nectaries suggest that *Burmannia* is pollinated by insects. However, only scarce information about pollination and reproduction of this genus is currently available. Several species of *Burmannia* are known to perform pollinator-independent autogamy (mostly via cleistogamy), including *B. capitata* (Walter ex J.F.Gmel.) Mart. (Wood, 1983), *B. championii* (Ernst and Bernard, 1912), *B. lutescens* [as *B. candida* (Blume) Engl., nom. illeg.] (Ernst and Bernard, 1912) and *B. wallichii* (Miers) Hook.f. (Zhang and Saunders, 2000). *Burmannia coelestis* is reported to be apomictic (Ernst, 1909). The only direct indications of entomophily in *Burmannia* are those published by Kato (1996) and Momose et al. (1998). These authors observed *B. lutescens* in Sarawak, Borneo, visited by two genera of mosquitoes (Culicidae, Diptera) whose proboscises correspond to the width of the openings between stigma and stamens and to the depth of the floral tube (Kato, 1996). However, it remains unclear, whether mosquitoes are the regular pollinators of this species, and do the pollinators facilitate xenogamy, geitonogamy or autogamy in *Burmannia*.

Our study indicates a need for broad-scale investigations of reproductive biology in Burmanniaceae. First, we failed to reveal any structural characters of flowers and inflorescences correlating with the mode of nutrition (mycoheterotrophic vs. photosynthetic lifestyle). Our data therefore suggest uniform pattern of pollination biology in *Burmannia*, which would contradict the idea of a “mycoheterotroph floral syndrome” (Waterman et al., 2013, p. 281), i.e., a putative set of adaptive floral features common to mycoheterotrophic angiosperms due to their common ecological preferences (Leake, 1994; Waterman et al., 2013). This question is to be resolved by direct observations of pollination process in species with different mode of nutrition. Second, it appears worthwhile to compare the patterns of reproductive ecology in the species of *Burmannia* with and without a gynostegium, including the diversity of floral visitors and their interactions with flowers, as the presence/absence of a gynostegium is possibly of high adaptive significance.

## Conclusion

Investigation of *Burmannia* that employed a representative species sampling allowed to obtain a comprehensive picture of the structural diversity of the flowers and inflorescences in the genus and to clarify a set of morphological characters, which are important for taxonomic delimitation and identification of the species, as well as for various evolutionary reconstructions.

A basic (most probably, ancestral) type of inflorescence in *Burmannia* is a thyrsoid with two lateral monochasial cymes. The cymes usually correspond to cincinni. In few-flowered species, the inflorescence is often a botryoid. The botryoids have possibly evolved from the thyrsoids via reduction of lateral cymes. The difference between the two types of inflorescences in *Burmannia* is quantitative rather than qualitative; in some few-flowered species, scarce branching occurs, which makes such inflorescences closer to thyrsoids.

The floral tube in *Burmannia* is a complex structure, consisting of a hypanthium and a perianth tube. Since the stamens are attached close to the apex of the floral tube, the perianth tube is much shorter than the hypanthium, and it is hardly recognizable in mature flowers of some species. The so-called perianth wings or ribs are solid structures, but morphologically each wing consists of three portions belonging to three floral parts: the ovary wall, the hypanthium and the perianth tube.

We demonstrated the presence of the inner tepal lobes in *B. oblonga*, a species that was believed to exhibit their complete absence. In this species, the inner tepal lobes initiate in the same manner as in the other studied species and remain visible (although very short) up to middle developmental stages of the flower. The extreme degree of reduction of the inner tepals in *B. oblonga* is accompanied by the absence of their vascular bundles in the floral tube. In *B. lutescens* and *B. itoana*, which are characterized by small definitive inner tepal lobes, the bundles of these lobes are present in the floral tube, but terminate below the level of the lobe attachment, leaving the lobes unvascularized. We conclude that the genus *Burmannia* is characterized by a uniform floral groundplan and shows a tendency toward reduction of the inner tepal lobes up to their apparent absence at maturity. Within this view, the apparent variation of the absence/presence of the inner tepal lobes represents a variation of the size of the lobes.

The ovary of *Burmannia* consists of a long symplicate zone and a much shorter synascidiate zone. In most species, the symplicate zone is secondarily trilocular throughout its length, whereas in the others the symplicate zone is trilocular proximally and unilocular distally. The intrusive Y-shaped placentas occupy the entire symplicate zone and a short distal part of synascidiate zone. The ovary roof and the common style are formed by postgenitally fused distal parts of carpels and represent the asymplicate zone. The distal region of the ovary contains septal nectaries that open via slits at the ovary roof. The lines of postgenital fusion in symplicate and asymplicate zones are indiscernible in mature gynoecia, which complicates recognition of gynoecial zones in individual cross sections.

The orifice of the floral tube is occluded by the deeply synorganized stamens and the distal portion of the common style. In some species, the synorganization is limited to the tight disposition of these organs, whereas in the others a gynostegium is formed by a postgenital fusion between the stamen connectives and the common style.

## Data Availability Statement

The original contributions presented in the study are included in the article/Supplementary Material, further inquiries can be directed to the corresponding author/s.

## Author Contributions

SY was involved in fieldwork, sectioning, SEM studies, microscopy, assembled the figures, and drafted the manuscript. AK was involved in conceptualization and collected plant material. BT, NV, and DL collected the plant material and investigated the floral morphology. MN was involved in fieldwork, preparation of the original draft and figures, conceptualization, and interpretation. MR was involved in sectioning, figure preparation, conceptualization, and interpretation. All the authors reviewed drafts of the manuscript, and approved the final version.

## Conflict of Interest

The authors declare that the research was conducted in the absence of any commercial or financial relationships that could be construed as a potential conflict of interest.

## Publisher’s Note

All claims expressed in this article are solely those of the authors and do not necessarily represent those of their affiliated organizations, or those of the publisher, the editors and the reviewers. Any product that may be evaluated in this article, or claim that may be made by its manufacturer, is not guaranteed or endorsed by the publisher.
