# Supplementary material for: Structure and Development of Flowers and Inflorescences in Burmannia (Burmanniaceae, Dioscoreales)
Source: Front Plant Sci. 2022 Mar 18;13:849276. doi: 10.3389/fpls.2022.849276 (PMC8971816; doi:10.3389/fpls.2022.849276)
Supplement: Supplementary file 1 [file Data_Sheet_1.PDF]

Supplementary Material

Supplementary Figures

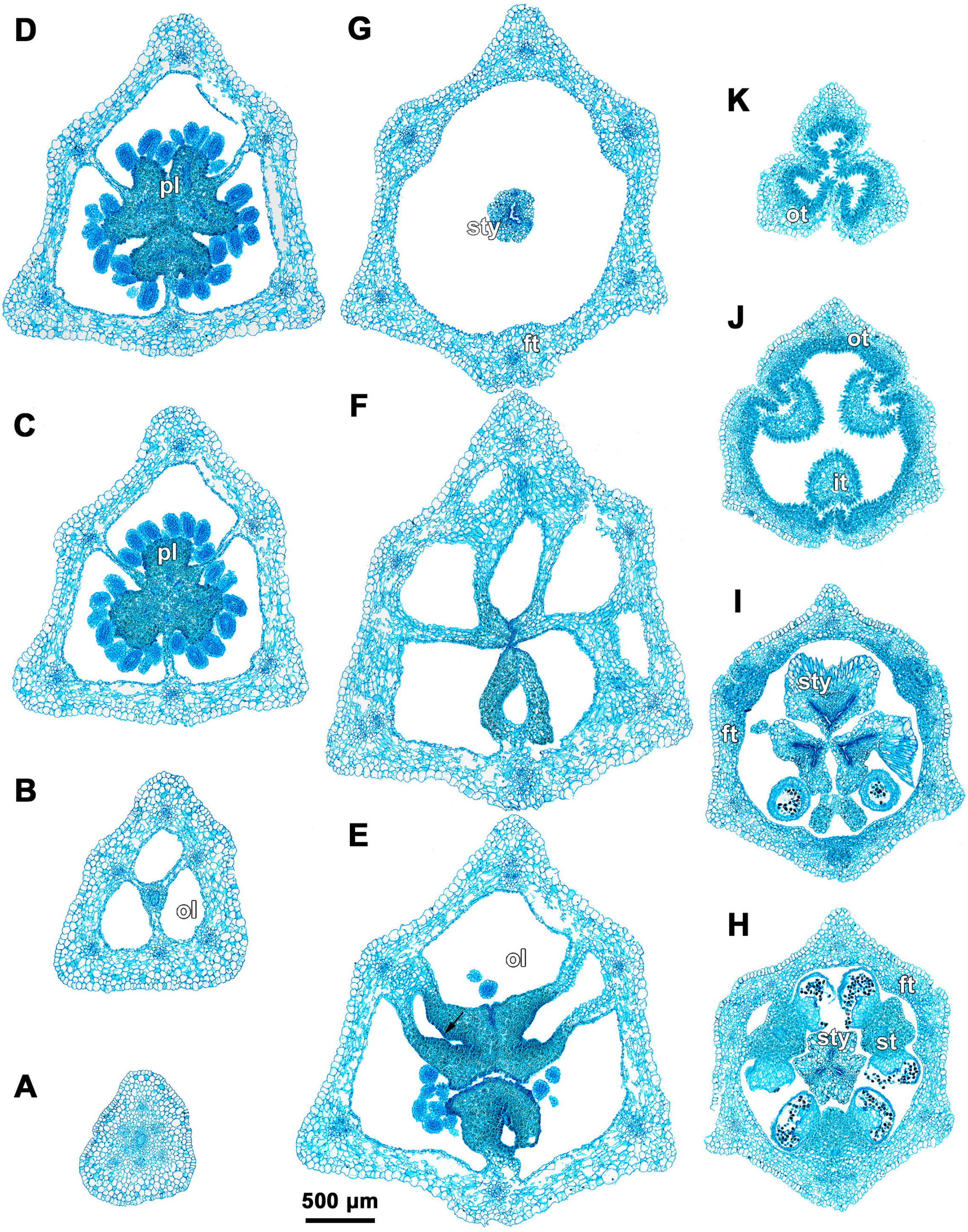

Supplementary Figure 1. Ascending series of transverse microtome sections of flower bud of *Burmannia championii* (LM).

(A) Pedicel. **(B)** Synascidiate zone of ovary. **(C)** Cross zone of ovary. **(D)** Symplicate zone of ovary (secondarily trilocular). **(E)** Symplicate zone of ovary just below the ovary roof. **(F)** Ovary roof. **(G)** Flower at level of common style. **(H)** Flower at level of anthers and common style. **(I)** Flower at level of stigmas. **(J)** Outer and inner tepal lobes. **(K)** Outer tepal lobes. Arrow indicates septal nectary. ft = floral tube; it = inner tepal; ol = ovary locule; ot = outer tepal; pl = placenta; st = stamen; sty = style.

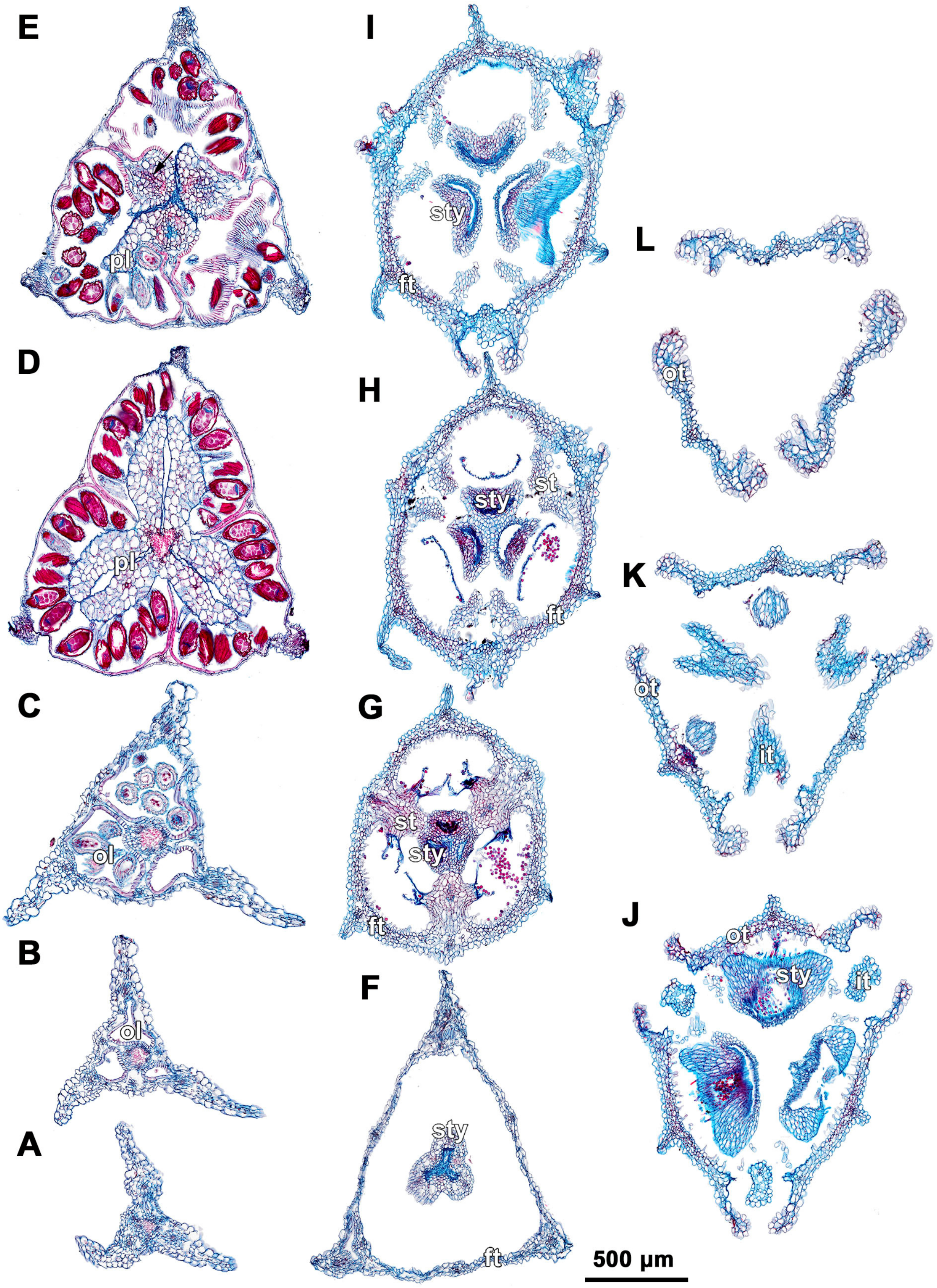

Supplementary Figure 2. Ascending series of transverse microtome sections of flower bud of *Burmannia chinensis* (LM).

(A) Pedicel just below ovary. **(B, C)** Synascidiate zone of ovary. **(D)** Symplicate zone of ovary (secondarily trilocular). **(E)** Symplicate zone of ovary just below the ovary roof. **(F)** Flower at level of common style. **(G)** Flower at level of gynostegium. **(H, I)** Flower at level of apical crests of stamens. **(J)** Flower at level of perianth lobes and stigmas. **(K)** Outer and inner tepal lobes. **(L)** Outer tepal lobes. Arrow indicates septal nectary. ft = floral tube; it = inner tepal; ol = ovary locule; ot = outer tepal; pl = placenta; st = stamen; sty = style.

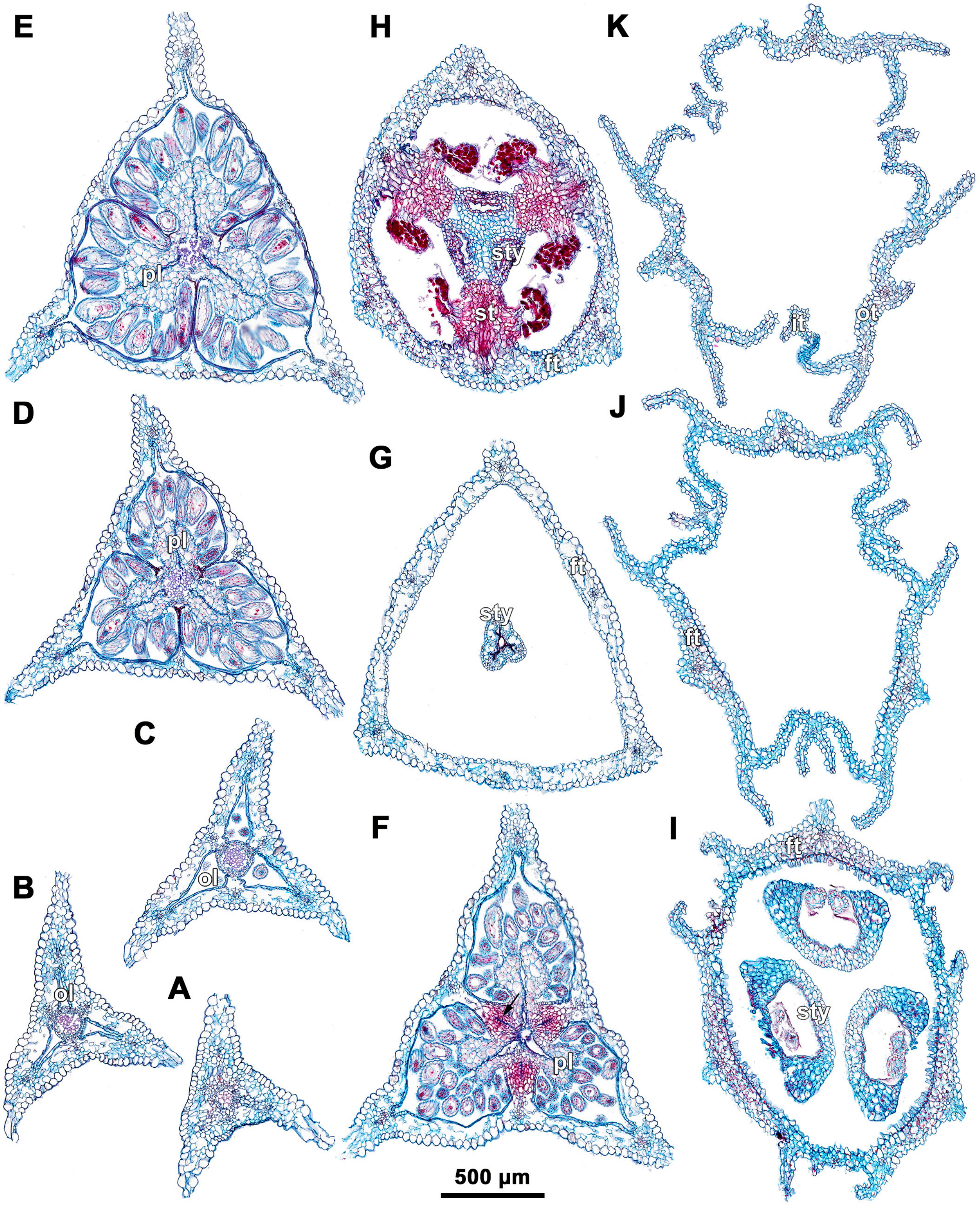

Supplementary Figure 3. Ascending series of transverse microtome sections of flower bud of *Burmannia coelestis* (LM) (*Nuraliev et al. 2736*).

(A) Pedicel just below ovary. **(B, C)** Synascidiate zone of ovary. **(D, E)** Symplicate zone of ovary (secondarily trilocular). **(F)** Symplicate zone of ovary just below the ovary roof. **(G)** Flower at level of common style. **(H)** Flower at level of anthers and common style. **(I)** Flower at level of stigmas. **(J)** Perianth tube. **(K)** Outer and inner tepal lobes. Arrow indicates septal nectary. ft = floral tube; it = inner tepal; ol = ovary locule; ot = outer tepal; pl = placenta; st = stamen; sty = style.

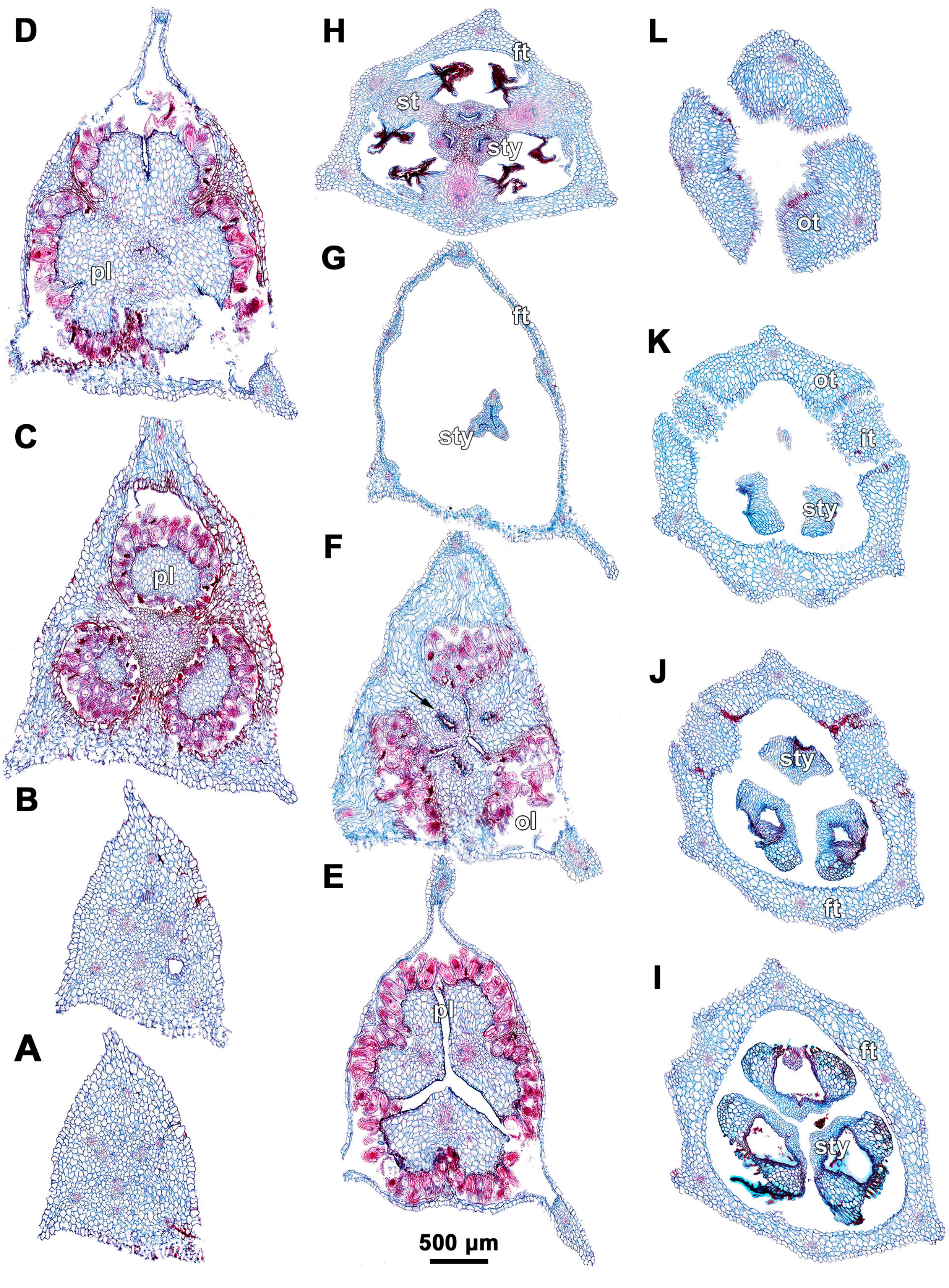

Supplementary Figure 4. Ascending series of transverse microtome sections of flower bud of *Burmannia itoana* (LM).

(A) Pedicel just below ovary. **(B)** The very base of synascidiate zone of ovary. **(C)** Synascidiate zone of ovary. **(D)** Secondarily trilocular portion of symplicate zone of ovary. **(E)** Distal (unilocular) portion of symplicate zone of ovary. **(F)** Symplicate zone of ovary just below the ovary roof. **(G)** Flower at level of common style. **(H)** Flower at level of gynostegium. **(I, J)** Flower at level of stigmas. **(K)** Outer and inner tepal lobes. **(L)** Outer tepal lobes. Arrow indicates septal nectary. ft = floral tube; it = inner tepal; ol = ovary locule; ot = outer tepal; pl = placenta; st = stamen; sty = style.

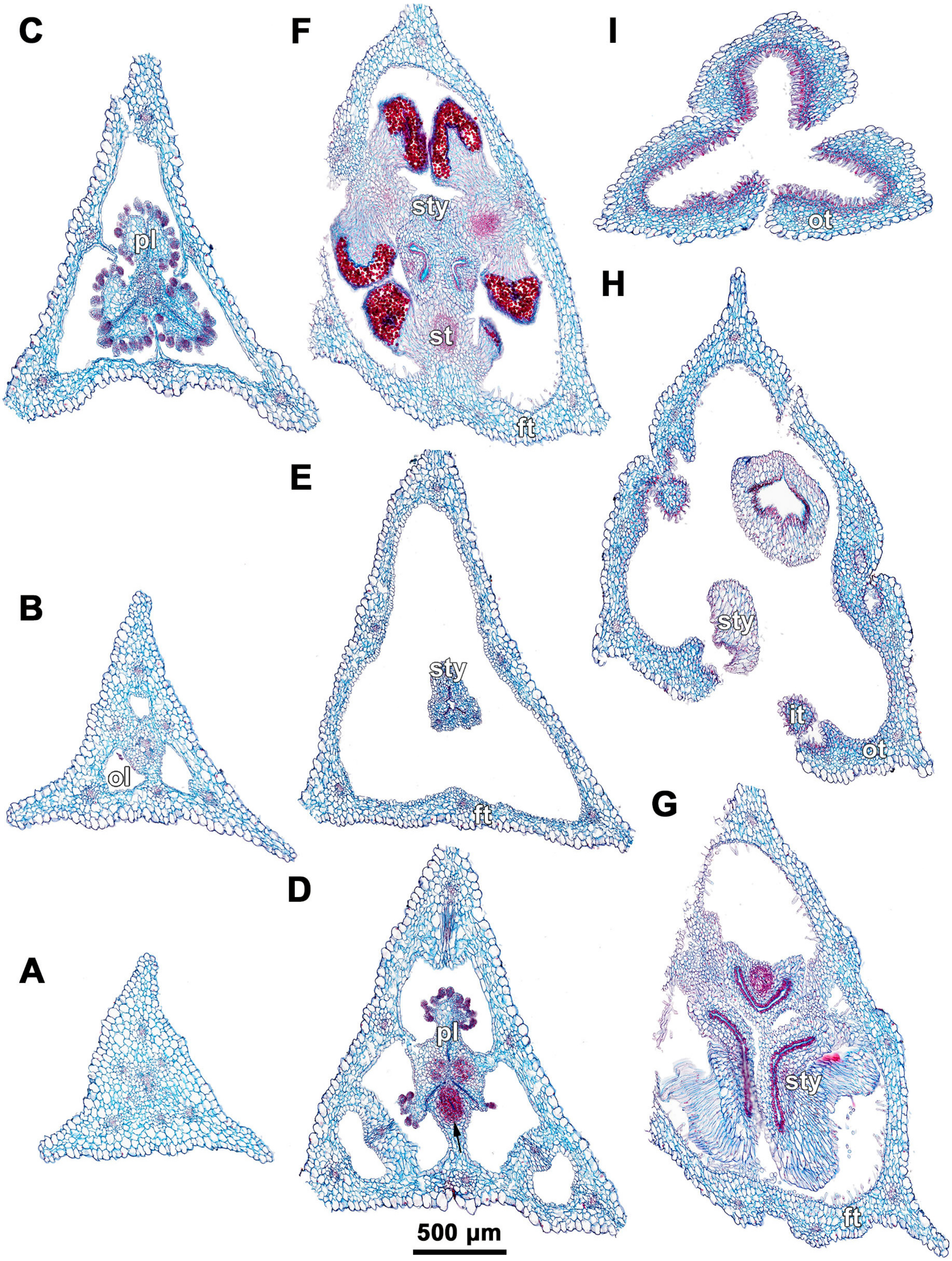

Supplementary Figure 5. Ascending series of transverse microtome sections of flower bud of *Burmannia lutescens* (LM) (*Nuraliev, Lyskov NUR 3120*).

(A) Pedicel just below ovary. **(B)** Synascidiate zone of ovary. **(C)** Symplicate zone of ovary (secondarily trilocular). **(D)** Symplicate zone of ovary just below the ovary roof. **(E)** Flower at level of common style. **(F)** Flower at level of gynostegium. **(G)** Flower at level of stigmas. **(H)** Flower at level of perianth lobes. **(I)** Outer tepal lobes. Arrow indicates septal nectary. ft = floral tube; it = inner tepal; ol = ovary locule; ot = outer tepal; pl = placenta; st = stamen; sty = style.

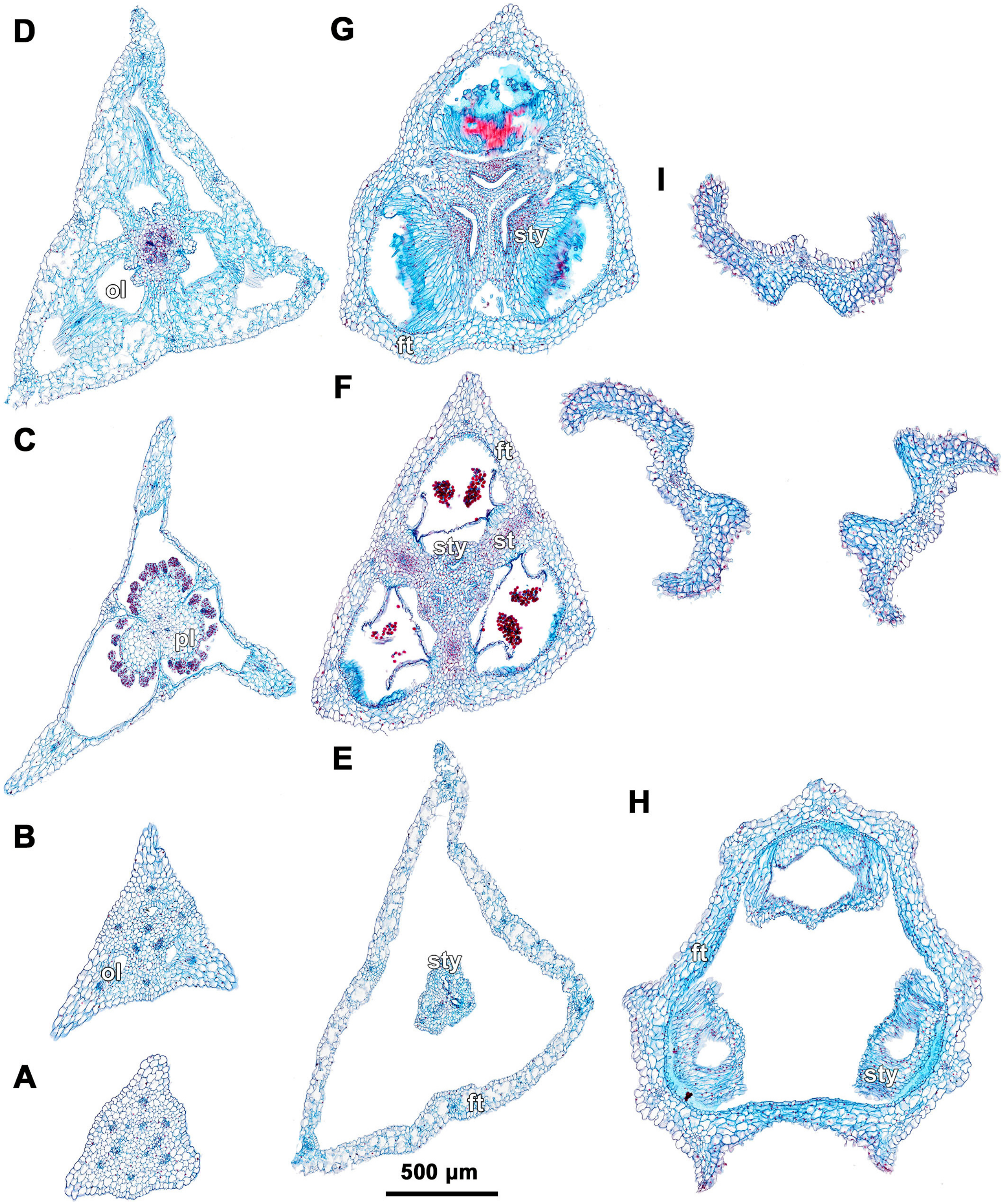

Supplementary Figure 6. Ascending series of transverse microtome sections of flower bud of *Burmannia oblonga* (LM) (*Poyarkov s.n.*).

(A) Pedicel just below ovary. **(B)** The very base of synascidiate zone of ovary. **(C)** Distal portion of synascidiate zone of ovary. **(D)** Ovary roof. **(E)** Flower at level of common style. **(F)** Flower at level of gynostegium. **(G, H)** Flower at level of stigmas. **(I)** Tepal lobes. ft = floral tube; ol = ovary locule; pl = placenta; st = stamen; sty = style; t = tepal.

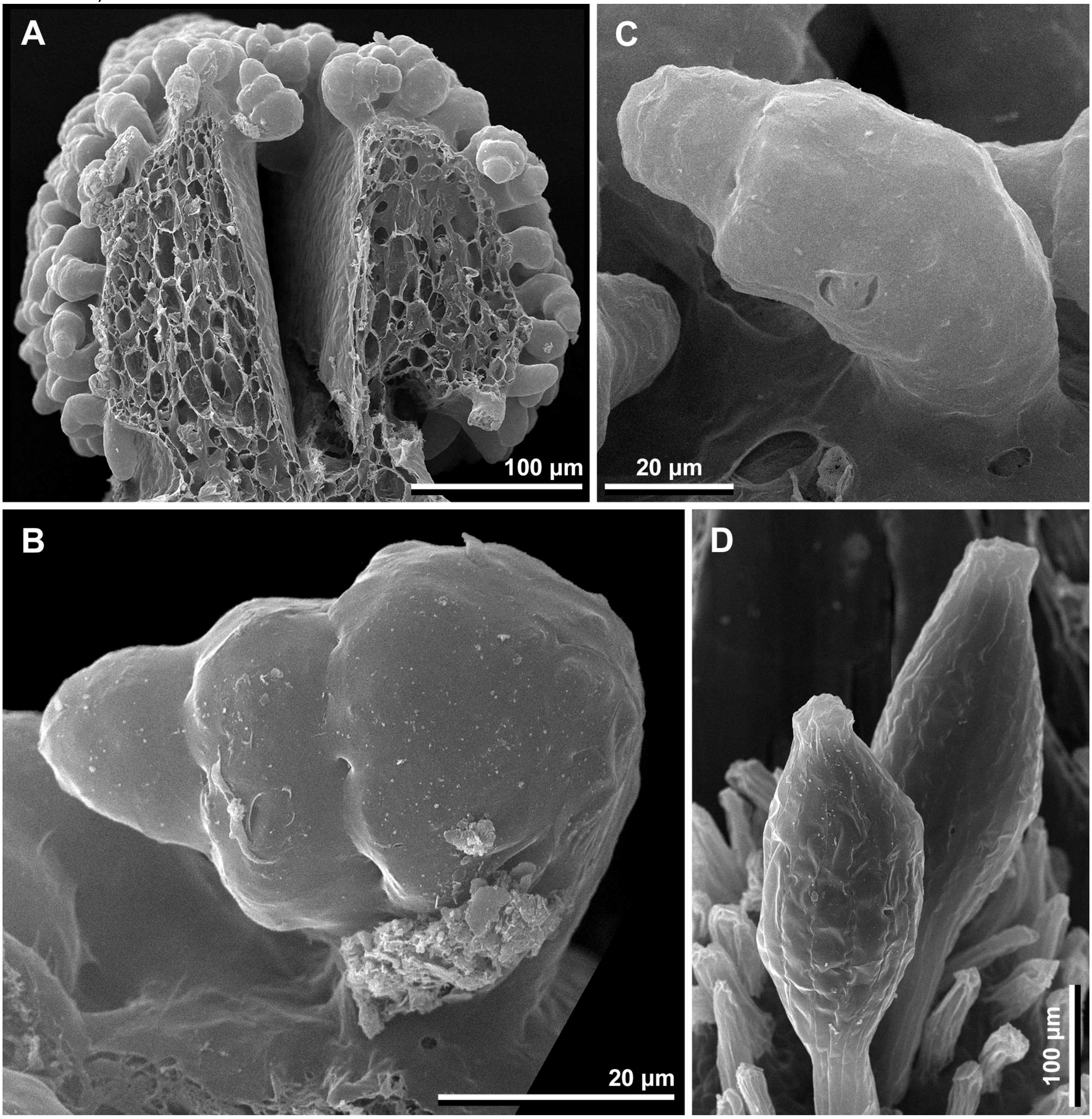

Supplementary Figure 7. Development of placentas and ovules of *Burmannia lutescens* (SEM).

(A) Placenta in symplicate zone, cross section. **(B)** Ovule from (A), showing initiation of two integuments. **(C)** Ovule at a later stage than that in (B). **(D)** Nearly mature ovule. A,B,D: *Nuraliev, Lyskov NUR 3120*; C: *Nuraliev et al. 1657*.
